# CircGCN1L1 promotes synoviocyte proliferation and chondrocyte apoptosis by targeting miR-330-3p and TNF-α in TMJ osteoarthritis

**DOI:** 10.1038/s41419-020-2447-7

**Published:** 2020-04-24

**Authors:** Huimin Zhu, Yihui Hu, Chuandong Wang, Xiaoling Zhang, Dongmei He

**Affiliations:** 1 Shanghai Key Laboratory of Stomatology & Shanghai Research Institute of Stomatology, National Clinical Research Center of Stomatology, Department of Oral Surgery, Shanghai Ninth People’s Hospital, Shanghai Jiao Tong University School of Medicine, Shanghai, China; 2 Department of Orthopedic Surgery, Xin Hua Hospital Affiliated to Shanghai Jiao Tong University School of Medicine (SJTUSM), Shanghai, China

**Keywords:** Mechanisms of disease, Long non-coding RNAs

## Abstract

Altered expression of circular RNAs (circRNAs) has been identified in various human diseases. In this study, we investigated whether circRNAs function as competing endogenous RNAs to regulate the pathological process of temporomandibular joint osteoarthritis (TMJOA). High-throughput sequencing of mRNA (RNA seq) was performed to detect the expression of circRNAs in TMJOA and control synovial tissues isolated from humans. The differentially upregulated circGCN1L1 (hsa_circ_0000448) in synoviocyte was validated in vitro and in vivo. Here we demonstrate the interactions between circGCN1L1 and both miR-330-3p and tumor necrosis factor-α (TNF-α) through bioinformatics predictions, luciferase report assays, and fluorescence in situ hybridization. mRNA expression profiles of TNF-α-stimulated synoviocyte showed that circGCN1L1 and p65 expressions were upregulated by TNF-α. Moreover, miR-330-3p was negatively correlated with TNF-α secretion. Further, we found that miR-330-3p directly targeted TNF and restrained the production of matrix-degrading enzymes (MMP3, MMP13, and ADAMTS4). Mechanistic studies unveiled that circGCN1L1 in TMJOA synovial tissues and cells may be associated with condylar chondrocyte apoptosis and synoviocyte hyperplasia. Moreover, intra-articular injection of shcircGCN1L1 alleviated TMJOA progression in rat models. Altogether, we elucidated the important roles of a novel circRNA, namely, circGCN1L1, which induced inflammation in TMJ synoviocytes and decreased anabolism of the extracellular matrix (ECM) through miR-330-3p and TNF-α gene. This circRNA may represent a potentially effective therapeutic strategy against TMJOA progression at an early stage.

## Introduction

Temporomandibular joint (TMJ) osteoarthritis (OA) is one of the most common temporomandibular joint diseases and is characterized by joint clicking/noise, pain, and restricted mouth opening and mandible movements, which may affect sufferers physically or mentally and their daily activities^1^. As a motivating factor of TMJOA, TMJ synovitis could activate inflammation throughout the joint, resulting in cartilage degradation and subchondral bone resorption^2,3^. The molecular mechanisms inducing the joint inflammation that leads to TMJOA remain unclear, and clinical treatment effects for TMJOA were limited. Hence, elucidating these mechanisms may facilitate the discovery of more effective therapies for TMJOA that delay or halt its progression at an early stage^4,5^.

Researchers consider circRNAs key regulators of the inflammatory response induced by osteoarthritis and potential new therapeutic targets in OA^6–9^. Currently, little is known about the functional roles of circRNAs in TMJOA, particularly in the TMJOA synovium. Some circRNAs were recently shown to be enriched in miRNA-binding sites and to naturally sequester miRNAs and competitively inhibit miRNA activity^10^. Interestingly, the existence of circRNAs in OA joint tissue (mainly knee joint tissue) was reported by Liu et al.^11^, who identified 71 differentially expressed circRNAs in human OA relative to normal cartilage. Xiang et al.^12^ also detected 122 differentially expressed circRNAs in the OA synovium. However, current research^13,14^ on synovial inflammation is focused mainly on limb joints and has not examined the mechanism or function of circRNAs in synovial tissue. Numerous studies have confirmed a high prevalence of synovial inflammation that persists through all stages of OA^15–17^. Researchers now recognize that joint synovial lesions potentially play pivotal roles in the pathogenesis of osteoarthritis. Here, we hypothesize that circRNAs may be central regulators of the TMJOA course.

In this study, we identified a specific circRNA (circGCN1L1) that is closely related to TMJOA using RNA deep-sequencing technology^18^ and systemically investigated its roles in vitro and in vivo.

## Methods

### Human TMJ synovial samples

The collection of all specimens was authorized by the patients and approved by the Ethics Committee of Shanghai Ninth People’s Hospital (Shanghai, China). Informed consent forms were signed before TMJ disc repositioning surgery. All samples were collected aseptically during the operation. Control synovial tissues were harvested from patients with anterior disc displacement in Wilkes Stage 2 (*N* = 20: 5 for RNA seq, 10 for vitro real-time (RT) quantitative polymerase chain reaction (qPCR) validation, and 5 for vitro cell culture and other experiments). The pathological synovial tissues were acquired from patients with TMJOA (Wilkes Stage 4) based on clinical symptoms and magnetic resonance imaging (MRI) diagnoses (*N* = 20: 5 for RNA seq, 10 for vitro RT-qPCR validation, and 5 for vitro cell culture and other experiments). Basic information and clinical characteristics of the patients are listed in Supplementary Tables 1S1 and 1S2.

### Human TMJ synoviocyte culture

The synovium was washed with sterile phosphate-buffered saline (PBS) three times, then cut into approximately 1–2-mm^3^ sections, and rinsed with Dulbecco’s modified Eagle’s medium (DMEM) containing 20% fetal bovine serum (FBS, Thermo Fisher Scientific, Waltham, MA, USA) until the solution was clear. The pieces were arranged on the bottom of the dish and incubated in a 37 °C incubator with 5% CO_2_ for 4 h. Pre-warmed DMEM containing 20% FBS was added into the dish to cover the tissue (with efforts made to avoid disturbing the dish, including limiting the numbers of observations and medium changes and avoiding shaking). The blocks were cultivated until synoviocytes migrated from the tissue.

### Human TMJ condylar chondrocyte culture

Condylar cartilage samples were isolated from three patients who had a TMJ condylar fracture (*N* = 3). TMJ condylar chondrocytes were cultured as described in our previous study^19^. The clinical characteristics of those patients are listed in Supplementary Table 1S3.

### RNA seq

Total RNA was extracted from five TMJOA tissues and five control tissues using Trizol reagent (Invitrogen, Carlsbad, CA, USA) according to the manufacturer’s protocol. The RNA concentration, purity, and integrity of each sample were quantified, and then the RNA library (mRNA and circRNA) was constructed using the methods described by Shen et al.^20^. The RNA libraries were then sequenced by Cloud-seq Biotechnology (Shanghai, China). Paired-end reads were obtained from an Illumina HiSeq 4000 sequencer and subjected to quality control using Q30. After trimming 3′ adaptors and removing low-quality reads using cutadapt (https://cutadapt.readthedocs.io/), the high-quality trimmed reads were used to analyze the expression of circRNAs and mRNAs.

### Analysis of mRNA expression

The high-quality reads were aligned to the human reference genome (UCSC hg19) with HISAT 2 software (http://ccb.jhu.edu/software/hisat2/index.shtml). Then, guided by the Ensembl gtf gene annotation file, cuffdiff software (http://cole-trapnell-lab.github.io/cufflinks/cuffdiff/) was used to obtain the FPKM values as the expression profiles of mRNA, and fold changes and *p* values were calculated based on the FPKM values. This progress was conducted under the guidance of Cloud-seq Biotechnology (Shanghai, China).

### Bioinformatics analysis of related RNA-seq data

#### Prediction of circRNA–miRNA interactions

The miRNA targets of circGCN1L1 were predicted using three different databases: circRNA-Interactome (https://circinteractome.nia.nih.gov/), StarBase (http://starbase.sysu.edu.cn/), and RegRNA2.0 (http://regrna2.mbc.nctu.edu.tw/). After selecting the results with a high level of evidence based on their indexes, the overlapping interactions were presented as a Venn diagram constructed using a web-based tool (http://bioinformatics.psb.ugent.be/webtools/Venn/).

#### Prediction of mRNA–miRNA interactions

The miRNAs targeting the TNF gene (the gene encoding the key inflammatory cytokine tumor necrosis factor-α (TNF-α) in TMJOA) were predicted using three different databases: TargetScan (http://www.targetscan.org/vert_72/), StarBase (http://starbase.sysu.edu.cn/), and miRanda (http://www.microrna.org/microrna/home.do).

#### Gene set enrichment analysis

Gene set enrichment analysis (GSEA) (using hallmark gene set: h.all.v6.2.symbols.gmt) was used for analysis (http://software.broadinstitute.org/gsea/downloads.jsp) according to the manufacturer’s protocol.

### RNA extraction and RT-qPCR analysis

Total RNA was extracted from cells and tissues using Trizol reagent. SYBR Premix Ex Taq II (TaKaRa) and Script RT reagent kit (TaKaRa) were used for analyses, and the reactions were subsequently measured on Roche LightCycler^®^ 480II PCR instrument (Basel, Switzerland). RT-qPCR was conducted from reverse transcription to amplification reactions using the methods described by Shen et al. MiRNA Isolation Kit (Thermo Fisher Scientific, Waltham, MA, USA) was used to extract the miRNAs. For the circRNAs, specific primers crossing the back-spliced junction were designed, and RT-qPCR was performed without the RNase R treatment. All reactions were analyzed in triplicate and normalized to the housekeeping gene U6 for miR-330-3p and GAPDH/ACTB (β-actin) for mRNAs and circRNAs. All the primers are listed in Supplementary Table 2. The relative mRNA/miRNA/circRNA expression levels were calculated using the 2^−^^ΔΔCt^ method.

### Sanger sequencing of RT-qPCR products for circRNAs

The amplification products were detected using agarose gel electrophoresis and Sanger sequencing with the appropriate protocols. The sequencing results were analyzed using Chromas software (http://technelysium.com.au/wp/chromas/) to identify the back-spliced junctions of specific circRNAs.

### CircRNA fluorescence in situ hybridization

CircRNA fluorescence in situ hybridization (FISH) assay was performed in TMJ synoviocytes from control patients. Cy3-labeled circGCN1L1 probes and reference Cy3-18sRNA (Ribo^TM^ h-18s FISH Probe Mix) and Cy3-U6 probes (Ribo^TM^ h-U6 FISH Probe Mix) were designed and synthesized by RiboBio (Guangzhou, China). The cell nucleus was labeled with DAPI (Sigma-Aldrich, St. Louis, MO, USA). The images were captured with a Nikon A1Si Laser Scanning Confocal Microscope (Nikon, Japan).

### CircGCN1L1 overexpression and knockdown

Both CircGCN1L1-overexpression vector and shRNA-expressing vector were purchased from Genomeditech (Shanghai, China). The entire circGCN1L1 (389 bp) sequence was included in circRNA-overexpression vector. *Bam*HI and *Eco*RI sites were inserted in the overexpression vector GPLVX-Laccase2-circGCN1L1-Puro through a double-restriction enzyme digest. The shcircGCN1L1 vector was constructed by pGMLV-SC5 RNAi lentivirus vector. Lipofectamine 3000 transfection reagent (Thermo Fisher Scientific, Waltham, MA, USA) was used for plasmid transfection. The sequences and primers are shown in Supplementary Table 2 and Supplementary Figs. 1 and 2.

### MiR-330-3p interference and overexpression

Specific miR-330-3p mimics and inhibitor were designed by Genomeditech (Shanghai, China) and transfected respectively into synoviocytes with Lipofectamine RNAiMAX transfection reagent (Thermo Fisher Scientific, Waltham, MA, USA). Sequences of miR-330-3p mimics and inhibitor are shown in Supplementary Table 2.

### Luciferase assay

HEK-293T cells purchased from Procell Life Science & Technology Co., Ltd. (Wuhan, China), which have been tested for mycoplasma contamination were seeded in 96-well plates and cultured to 70% confluence before transfection. For circGCN1L1 and miR-330-3p experiment, 100 ng of circGCN1L1 wild type (WT) and mutant type (MT), circRNA normal control (NC), and 30 nM miR-330-3p were transfected. For TNF and miR-330-3p, 100 ng of plasmids of TNF 3′UTR WT and TNF 3′UTR MT, 30 nM miR-330-3p, and NC were transfected. Each group was replicated in three parallel wells of a 96-well plate. After 48 h of incubation, firefly and *Renilla* luciferase activities were detected using a Luciferase Assay Kit (Genomeditech, Shanghai, China). Luciferase Assay Reagent II (LAR II) (Luciferase Assay Reagent, Promega) and lysis buffer were used subsequently. *Renilla* luciferase activities served as an internal reference, and Luc firefly/*Renilla* (termed Luc/Rena) ratios were calculated to determine relative luciferase activity. Lipofectamine™ 2000 (Invitrogen) was used for transfection. The vector information and sequences are listed in Supplementary Table 2 and Supplementary Fig. 3.

### Enzyme-linked immunosorbent assays

Transfect miR-330-3p mimics or inhibitor with different concentrations separately into human synoviocytes from the control patients. Collect the cell supernatant at different time points (1, 2, 4, 8, 16, 32, and 48 h). The secretion of the TNF-α protein was subsequently detected as described in the TNF-α ELISA Kit (Multiscience, Hangzhou, China) with the sensitivity of 0.16 pg/ml. A standard curve ranging from 1.56 to 100 pg/ml was generated using known concentrations of the respective purified recombinant TNF-α. Three parallel duplicates were analyzed at each time point.

### Flow cytometry assays

Condylar chondrocytes that co-cultured with synoviocytes were collected using trypsin without EDTA (Thermo Fisher Scientific, Waltham, MA, USA) and washed with PBS. Apoptosis was detected using the Annexin V-FITC/PI Apoptosis Kit (BD Biosciences, Franklin Lakes, NJ, USA) according to the manufacturer’s instructions. Apoptosis was detected with a BD FACS flow cytometer (BD Biosciences). For the cell cycle assay, cells were fixed in absolute ethanol overnight and washed twice, followed by incubation with a PI/RNase staining buffer (BD Biosciences). Finally, specimens were detected by a BD FACS (more than 10,000 events/sample were read) flow cytometer (BD Biosciences).

### Edu cell proliferation assay on synoviocytes and co-cultured chondrocytes

Transwell chambers with 0.4-μm pore size (Corning, USA) were used to establish the cell co-culture system. Primary chondrocytes were moderately digested with trypsin and inoculated into the lower chamber of a six-well Transwell plate. Synoviocytes from the control group were seeded in the upper Transwell chamber in another six-well plate for culture. The systems were divided into several groups (circGCN1L1, circGCN1L1 control, shcircGCN1L1, shcircGCN1L1 control, shcircGCN1L1 plus miR-330-3p, circGCN1L1 plus miR-330-3p inhibitor, and blank control). Transfect the plasmids above respectively into synoviocytes and then replace the medium 4–6 h later. Transfer the upper chamber back to the chondrocyte plate, and then co-culture the two kinds of cells for 2 days^21^. Single co-cultured synovial cells from the control patient were transferred from the same transfection as above. Edu cell proliferation assay was performed for lower chondrocytes and single-cultured synovial cells. Briefly, 48 h after transfection, pre-warmed 2× Edu working solution (RiboBio, Guangzhou, China) was added to the plate in equal volume, making the final Edu concentration 10 μM. Continue incubating the cells for 2 h. After that, 4% paraformaldehyde (PFA) fix solution was added. Wash the cells three times with PBS, then use 0.3% Triton X-100 permeate solution, and incubate at room temperature for 10–15 min. Add DAPI (Sigma-Aldrich, St. Louis, MO, USA) solution (1:1000) to each well, and incubate at room temperature for 10 min. Capture the images with a Nikon A1Si Laser Scanning Confocal Microscope (Nikon, Japan).

### Western blotting

Cells were lysed on ice for 30 min in lysis buffer containing 50 mM Tris-HCl, pH 7.4, 150 mM NaCl, 1% Nonidet P-40, and 0.1% SDS supplemented with protease inhibitors (10 mg/ml leupeptin, 10 mg/ml pepstatin A, and 10 mg/ml aprotinin). Protein fractions were collected by centrifugation at 10,000 × *g* for 10 min, separated on 10% SDS-PAGE gels, and then electrotransferred onto nitrocellulose membranes (Whatman, Piscataway, NJ, USA). The membranes were blocked with 5% BSA and then incubated with specific antibodies overnight at 4 °C. The primary antibodies and their sources were as follows: COL2A1 (1:1000, GB11021, Servicebio), ADAMTS4 (1:1000, ab185722, Abcam), p65 (1:5000, ab32536, Abcam), MMP3 (1:1000, ab52915, Abcam), MMP13 (1:1000, ab84594, Abcam), Caspase-7 (1:1000, DF6441, Affinity), cleaved Caspase-3 (1:1000, AF7022, Affinity), Caspase-3 (1:1000, AF6311, Affinity), Bcl-2 (1:2000, ab182858, Abcam), Bax (1:1000, ab32503, Abcam), TNF alpha (1:1000, ab1793, Abcam), β-actin (1:1000, ab6276, Abcam), and GAPDH (1:2000, AF7021, Affinity). HRP-conjugated secondary antibodies (ab205719 and ab6721, Abcam) were used at a 1:2000 dilution. The antigen–antibody complexes were visualized using an enhanced chemiluminescence detection system (Millipore, Darmstadt, Germany) according to the manufacturer’s instructions.

### Rat model of TMJOA

All rats were acquired from Central Laboratory of Shanghai Ninth People’s Hospital (Shanghai, China), and rat housing and welfare procedures were performed with the approval from the Institute of Health Sciences Institutional Animal Care and Use Committee (IACUC). A total of 40 female Sprague-Dawley (SD) rats (aged 4 weeks) weighing 160–200 g were divided into four groups (10 rats in each group). The animals were first divided into four blocks according to body weight, and then the animals in each block were randomly assigned to each group according to a random number table. A model of TMJOA was established by unilateral anterior occlusion induction as described previously^22^. Briefly, cut a No.20 teat cannula to a 8-mm-length tube using a low-speed hand saw. Use a needle holder to bend one side of the tube to 135° and is 3.5 mm in length. After 1% pentobarbital anesthesia (0.35 ml/100 g body weight), adhere this crown by zinc phosphate cement on the left lower incisor of the rat to induce the unilateral anterior occlusion change. Thus, the unilateral anterior occlusion model was established, wherein the left incisor was reversibly engaged and the right incisor was normal. Check the metal crown at least once a day to ensure that it remained in place. A sham induction was performed in the control group. Anesthetize the rat and apply zinc phosphate cement to lower the left incisor. After the treatment, free activity and normal diet were allowed in the cage. Thirty rats underwent unilateral anterior occlusion induction and the remaining 10 rats underwent sham induction.

### Articular injection of shcircGCN1L1 in rats

One week after the initial treatment, the unilateral anterior occlusion induction rats were randomly divided into three groups (occlusion-induced OA plus PBS, occlusion-induced OA plus shcircGCN1L1 injection, and occlusion-induced OA plus shcircGCN1L1 MT injection) with 10 rabbits in each group. The shcircGCN1L1 and shcircGCN1L1 MT plasmids were dissolved separately in PBS to generate a 500 ng/μl solution. A total of 20-μl solution was slowly injected into the left TMJ cavity of the rats. Rats in the control group were injected with PBS. The injection procedure was repeated once a week, and the rats were sacrificed after 4 weeks. The left TMJ condyle was used for histological analysis.

### Histological analysis, Safranin-O/Fast green staining, and OARSI score system

Rat condylar specimens were fixed with 4% paraformaldehyde and embedded in paraffin. Paraffin blocks were sectioned at a thickness of 7 μm. The sections were deparaffinized in xylene and dehydrated with a graded series of ethanol solutions. Each section was stained with hematoxylin–eosin (HE) and 0.1% Safranin-O solution and 0.001% Fast green solution (Sigma-Aldrich, St. Louis, MO, USA)^23^. Condylar cartilage destruction was scored by three observers blinded to experiment information using the OARSI grading system^24^. The OA damage grade was divided into six levels. Grade 1 would have normal chondrocytes. In grade 1.5, loss of Safranin-O and/or Fast green would be seen. In grade 2, small fibrillation formation without cartilage loss would be seen. In grade 3, abrasion of the superficial cartilage would be seen additionally. In grade 4, vertical clefts down to the layer below the superficial layer with some loss of surface lamina. In grade 5, fissures or erosion penetrating into the mid zone with intact calcified cartilage would be seen. In grade 6, the joint geometry deformity at the joint margins is seen, and the erosion penetrates into the calcified cartilage or subchondral bone. Four TMJOA stages were defined according to the involved cartilage surface, irrespective of the underlying OA grade. Stage 1 showed less than 10% involvement, whereas stage 2 showed 10–25% involvement. In addition, stage 3 showed 25–50% involvement, whereas stage 4 showed more than 50% involvement. The score is equal to the grade times the stage.

### Immunohistochemistry

Antigen retrieval was performed by incubating sections with 0.05% trypsin (pH 7.8) at 37 °C for immunohistochemistry (IHC). After being blocked with 1% BSA, the sections were incubated with primary antibodies against COL2A1 (1:200, GB11021, Servicebio), MMP3 (1:100, ab52915, Abcam), and MMP13 (1:100, ab84594, Abcam) at 4 °C overnight and then for 1 h at 37 °C with HRP-labeled secondary antibodies (Beyotime Biotechnology, Jiangsu, China). Then use DAB stain for 10 min until the brown color appeared. After hematoxylin counterstaining, 1% alcohol differentiation, and dehydration, use neutral gum to cover the slides. The positively stained cells were counted, and the percentage was calculated by Image-J 1.52t^25^.

### Statistical analysis

Statistical analyses was performed with SPSS v22.0 software. The distribution of the data was analyzed with the Shapiro–Wilk test, and the equality of variances was evaluated with Levene’s test. Statistical analyses were performed using the paired two-tailed Student’s *t-*test (normal distribution and equal variances, using 95% confidence intervals for between-group differences), Welch’s *t-*test (unequal variances), or the Mann–Whitney *U* test (non-normal distribution) for comparisons between groups. Comparisons among groups were performed using one-way analysis of variance (normal distribution) or the Kruskal–Wallis test (non-normal distribution) followed by the Bonferroni or Dunn post hoc test. For all analyses, data are presented as mean ± S.D., and group differences were considered significant at *p* < 0.05 (*p < 0.05).

### Statement of ethics

This study was approved by the Ethics Committee of the Shanghai Ninth People’s Hospital affiliated to Shanghai Jiaotong University. The collection of specimens was authorized by all patients, who provided written informed consent.

## Results

### CircGCN1L1 expression and localization in TMJ synovial tissues and cells

**Fig. 1:**
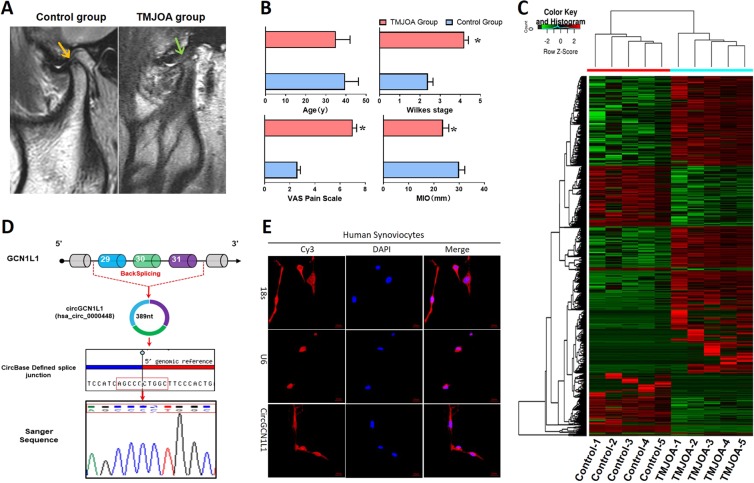
CircGCN1L1 expression and localization in TMJ synovial tissues and cells. **a** Representative MRI images of TMJOA and control patients. **b** Age, Wilkes Stage, MIO, and VAS pain score of TMJOA and control patients. *N* = 40 (20 different patients in each group). *p < 0.05. **c** Heat map of all differentially expressed circRNAs between TMJOA and control synovial samples. *N* = 10 (five different samples in each group). **d** Comparation of circGCN1L1 sequences acquired from circBase and UCSC Genome Browser with the GCN1L1 mRNA sequences. **e** Images of RNA FISH in synoviocytes from control patients. CircGCN1L1 probes were labeled with Cy3. Nuclei were stained with DAPI. Scale bar, 20 μm. U6 was used as an internal control for nuclear RNA, whereas 18s served as the control for cytoplasmic RNA. *N* = 3 (three different samples). Data are presented as mean ± S.D. Two-tailed *t-*test (**b**) was performed. MRI magnetic resonance imaging, TMJOA temporomandibular joint osteoarthritis, MIO maximum mouth incisor opening, VAS visual analog scale, FISH fluorescence in situ hybridization, DAPI dihydrochloride.

RNA seq was performed to analyze the ribosomal RNA-depleted total RNA from the synovial tissues of five patients with clinical TMJOA and five control patients with the aim of exploring the circRNA expression profiles in the TMJOA synovium. The MRI for patients with TMJOA presenting with significant anterior disc displacement without reduction, hydrarthrosis in the upper joint spaces, and bone resorption at the posterior slope of condyles and the control patients only with anterior disc displacement without reduction are shown in Fig. 1a. Patients with TMJOA were diagnosed with more severe Wilkes Stages, showed less maximum mouth-opening indexes, and higher visual analog scale for pain (VAS) scores in TMJ areas (Fig. 1b). Notably, 11,648 circRNAs were detected and identified by DCC (https://omictools.com/dcc-tool) after the high-quality reads were mapped to the reference genome/transcriptome (hg19, human genome). The identified circRNAs were annotated using the circBase database and Circ2Traits, 3384 of which had a circBase ID. The junction reads of each circRNA were normalized and subjected to Log2 conversion for comparisons between groups. The circRNA expression patterns were significantly different between the TMJOA and control groups (Fig. 1c), and are listed in Supplementary Table 3. The size of all differentially expressed circRNAs ranged from 300 to 6000 bp, and 7 of the top 10 were exonic-type circRNAs (Supplementary Table 1S4). Three circRNAs were upregulated in the TMJOA group (hsa_circ_0000605, FC = 167.12; hsa_circ_0000448 or circGCN1L1, FC = 153.3; chr21:37711073–37717005+, FC = 104.71) and the remaining seven were downregulated in the TMJOA group (hsa_circ_0009043, FC = 425.31; hsa_circ_0003154, FC = 71.01; hsa_circ_0000378, FC = 62.68; hsa_circ_0007444, FC = 55.09; chr17:33495080–33495704+, FC = 47.18; hsa_circ_0001801, FC = 46.02; hsa_circ_0001946, FC = 2.73). Given the high stability of circRNA^26^, we used RNase R to treat human synoviocyte to test whether it was true for the five circRNAs above. It is noteworthy that according to our previous study, circGCN1L1 was the most insensitive one to RNase R^18^. By comparing circGCN1L1 sequences acquired from circBase and UCSC Genome Browser with the GCN1L1 mRNA sequences, we determined that circGCN1L1 was looped and comprised exons 29–31 of its parental gene, whose head-to-tail splicing was further confirmed by Sanger sequencing (Fig. 1d). Moreover, circRNA FISH using a circGCN1L1-specific probe targeting the junction region was conducted. Quantitative analysis of the fluorescence intensity (Integrated Density, IntDen) in this FISH map shows that the fluorescence distribution ratio of the nucleus and cytoplasm is 0.214 (13.39:62.67/IntDen), demonstrating that circGCN1L1 predominantly (82.39%) localized in the cytoplasm of TMJ synoviocytes (Fig. 1e). The results above indicated that the abundant and stable expression of circGCN1L1 in the cytoplasm of TMJOA synoviocytes may have clinical significance for TMJOA.

### CircGCN1L1 serves as a sponge for miRNAs and targets miR-330-3p in human TMJ synoviocytes

**Fig. 2:**
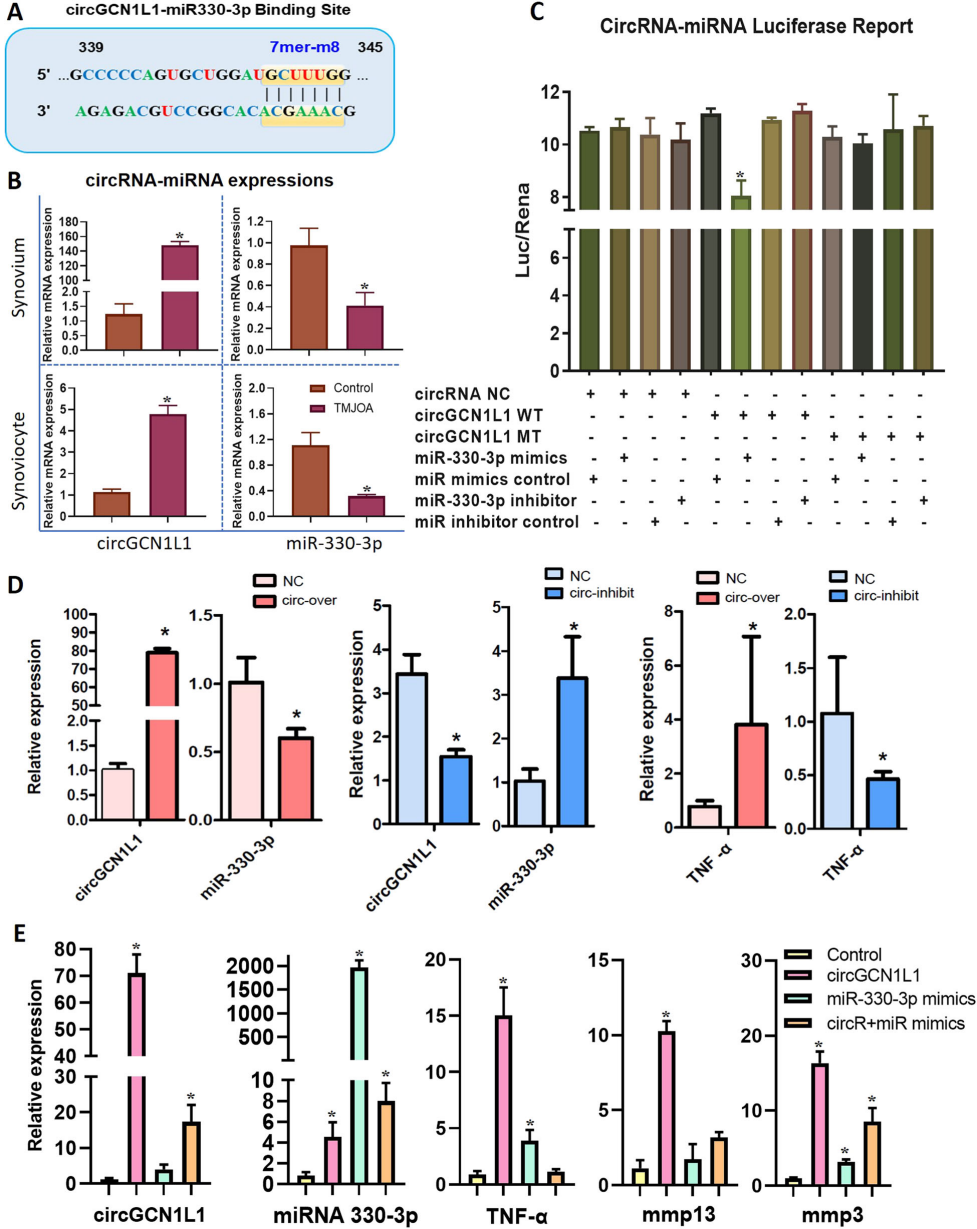
Prediction and validation of circGCN1L1-binding miRNAs. **a** Circular RNA Interactome, StarBase, and RegRNA2.0 databases were used to predict the interacting miRNAs of circGCN1L1 and its potential binding sites. **b** RT-qPCR detection of circGCN1L1 and miR-330-3p expression in synovial tissues and synoviocytes. *N* = 15 (five different samples in each group for three independent experiments). *p < 0.05. **c** HEK-293T cells were transfected with circGCN1L1 reporter or circGCN1L1 MT reporter and miR-330-3p or NC, followed by luciferase activity detection. Relative luciferase activity is demonstrated in the histogram. *N* = 3 (three replicates). *p < 0.05. **d** Synoviocytes from the control patient were transfected with 50 nM circGCN1L1 or shcircGCN1L1 plasmid. After 48 h, the expression levels of circGCN1L1 and TNF-α were measured by RT-qPCR and normalized to the β-actin level. The expression of miR-330-3p was measured by RT-qPCR and normalized to U6 expression. *N* = 6 (two different samples for three independent experiments). *p < 0.05. **e** Synoviocytes from the control patient were transfected with 50 nM circGCN1L1 plasmid and/or miR-330-3p mimics. After 48 h, the expression levels of circGCN1L1, TNF-α, MMP3, and MMP13 were measured by RT-qPCR and normalized to the β-actin level. The expression of miR-330-3p was normalized to U6 expression. *N* = 6 (two different samples for three replicates). *p < 0.05. Data are presented as mean ± S.D. Two-tailed *t*-test (**b**, **d**) and one-way ANOVA with Bonferroni test (**c**, **e**) were performed. TNF tumor necrosis factor, NC normal control, RT-qPCR real-time quantitative polymerase chain reaction.

CircRNAs typically function as miRNA sponges to indirectly regulate the expression of target genes^10^. As circGCN1L1 was abundantly expressed, stable, and closely related to TMJOA, we predicted the ability of circGCN1L1 to bind miRNAs using the Circular RNA Interactome, StarBase, and RegRNA2.0 databases. Although circGCN1L1 contains multiple miRNA-binding sites, miR-330-3p was the only miRNA identified by three databases (Supplementary Figs. 4 and 5). The 339th to 345th bases of circGCN1L1 matched exactly to miR-330-3p with the binding site type of 7mer-m8 (Fig. 2a). Therefore, miR-330-3p was selected for further investigation. RT-qPCR revealed that the expression of circGCN1L1 was upregulated in TMJOA synovial tissues and cells, whereas miR-330-3p expression was conversely downregulated in TMJOA synovial tissues and synoviocytes (Fig. 2b). Thus, circGCN1L1 and miR-330-3p showed an inverse relationship in TMJOA. The circRNA (circGCN1L1)-miRNA (miR-330-3p) luciferase assay indicated the direct binding of circGCN1L1 to miR-330-3p in HEK-293T cells (Fig. 2c). In vitro circGCN1L1 overexpression and inhibition systems were constructed in synoviocytes from control patients to demonstrate the relationship between circGCN1L1 and miR-330-3p (Fig. 2d). The knockdown efficiency and the expression of GCN1L1 were evaluated by RT-qPCR (Supplementary Fig. 6). In this experiment, miR-330-3p was downregulated after circGCN1L1 overexpression, whereas it was upregulated when circGCN1L1 was inhibited. In addition, synoviocytes from the control patient were transfected with 50 nM circGCN1L1 plasmid and/or miR-330-3p mimics. RT-qPCR results indicated that the expression of miR-330-3p in circGCN1L1 and miR-330-3p mimic-co-transfected group was lower than the miR-330-3p mimic-transfected group, whereas it was higher than the circGCN1L1-treated group. Interestingly, circGCN1L1 overexpression could have synergistic effects with much more severe ECM degradation phenotype, which is restricted after co-transfection of miR-330-3p mimics (Fig. 2e). These data suggested that circGCN1L1 may directly interact with miR-330-3p and could serve as a sponge for miRNAs in human TMJ synoviocytes.

### MiR-330-3p directly targets the TNF gene and regulates TNF-α expression and secretion

**Fig. 3:**
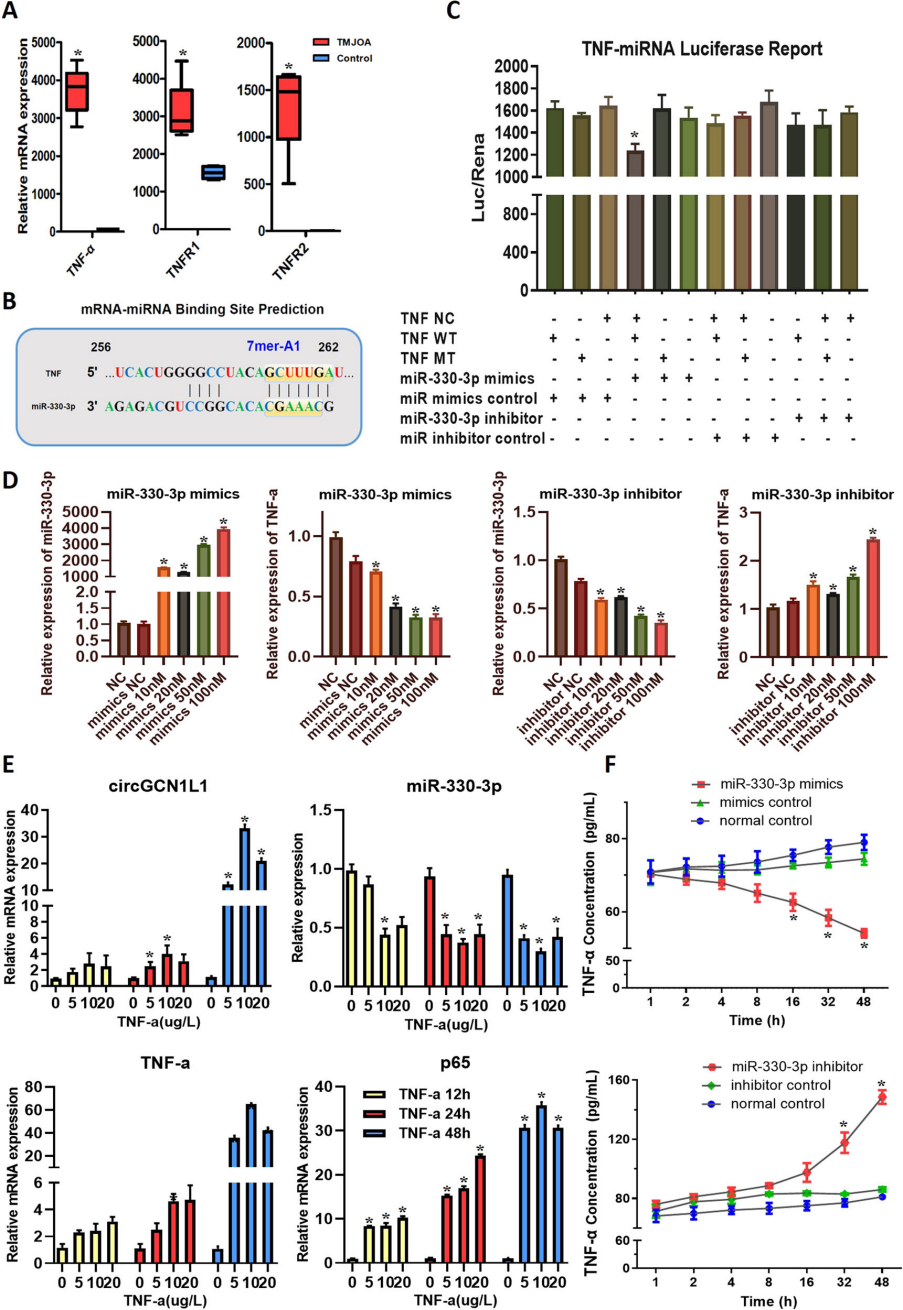
Expression of and relationships among circGCN1L1, miR-330-3p, and TNF-α in TMJ synovial tissues and cells. **a** TNF-α and its receptor (TNFR1 and TNFR2) expressions in human TMJOA synovial tissues and control tissue. *N* = 15 (five different samples in each group for three independent experiments). *p < 0.05. **b** TargetScan, miRanda, and StarBase databases were used to predicate the possible miRNAs targeting TNF. **c** HEK-293T cells were transfected with miR-330-3p or NC and luciferase reporter constructs containing MT or WT TNF 3′-UTR. *N* = 3 (one sample for three replicates). *p < 0.05. **d** Transfect miR-330-3p mimics or inhibitor into synoviocytes from the control patient at different concentrations (10, 20, 50, and 100 nM). *N* = 6 (two different samples for three independent experiments). *p < 0.05. **e** The mRNA levels of circGCN1L1, TNF-α, and p65 in synoviocytes from control patients stimulated with TNF-α at different doses (0, 5, 10, and 20 μg/l) for different time periods (12, 24, and 48 h) were measured by RT-qPCR and normalized to β-actin level. The expression of miR-330-3p was normalized to U6 expression. *N* = 6 (two different samples for three independent experiments). *p < 0.05. **f** Supernatants of synoviocyte from control patients transfected with miR-330-3p mimics or inhibitor were collected at different time points (1, 2, 4, 8, 16, 32, and 48 h), and the levels of TNF-α protein were detected using ELISA. *N* = 6 (two different samples for three independent experiments). *p < 0.05. Data are presented as mean ± S.D. Two-tailed *t-*test (**a**) and one-way ANOVA with Bonferroni test (**c**–**f**) were performed. TNF tumor necrosis factor, TNFR1 tumor necrosis factor receptor type 1, TNFR2 tumor necrosis factor receptor type 2, RT-qPCR real-time quantitative polymerase chain reaction, NC normal control, MT mutant, WT wide type, ELISA enzyme-linked immunosorbent assay.

Recent work has identified a battery of proteins that regulate processing (either positively or negatively) by binding to miRNA precursors^27^. The TNF gene plays a key role in TMJOA development, as previously reported^28,29^. Interestingly, TNF-α, one of the essential inflammation cytokines present at substantially higher levels in the TMJOA synovium than in the control synovium (Fig. 3a), displayed changes in expression that were consistent with the circGCN1L1 (Fig. 2e). In addition, the expression of TNF receptors (TNFR1 and TNFR2) was increased accordingly (Fig. 3a). Therefore, we investigated the possible miRNAs targeting the TNF gene by overlapping the predicted results of the TargetScan, miRanda, and StarBase databases (Supplementary Fig. 7). Notably, miR-330-3p targeted the TNF gene from the 256th to 262nd bases in the coding region with the binding site type of 7mer-A1 (Fig. 3b). The mRNA (TNF)-miRNA (miR-330-3p) luciferase assay results revealed that miR-330-3p mimics significantly inhibited luciferase activity in HEK-293T cells transfected with the TNF wild-type gene, but not in cells transfected with the mutant-type TNF gene (Fig. 3c). This finding indicated a direct binding of miR-330-3p to TNF. Therefore, we transfected different concentrations (10, 20, 50, and 100 nM) of miR-330-3p mimics/inhibitor and related controls into TMJ synoviocytes to evaluate the relationship between miR-330-3p and TNF-α. Four concentrations were applied, and the results indicate that TNF-α expression decreased accordingly as the concentration of miR-330-3p mimics increased, which is contrary to the outcome of miR-330-3p inhibition (Fig. 3d). Thereupon, we detected the mRNA levels of circGCN1L1, miR-330-3p, TNF-α, and p65 in synoviocytes from the control patient, which was stimulated with TNF-α at different doses (0, 5, 10, and 20 μg/l) for different time periods (12, 24, and 48 h). The results of RT-qPCR validation showed that the expression of circGCN1L1, TNF-α, and p65 was increased by TNF-α in time- and dose-dependent manners, and reached the highest at 10 μg/l of TNF-α for stimulation at 48 h. The TNF-α-induced inhibitory effect on miR-330-3p further verified the findings above (Fig. 3e). Besides, we collected the cell supernatant of synoviocytes, which transfected with miR-330-3p mimics/inhibitor (50 nM) described above at various time points (1, 2, 4, 8, 16, 32, and 48 h), and TNF-α protein levels were then detected using ELISA. The level of TNF-α secreted by synoviocytes did not change significantly with miR-330-3p overexpression during the first 8 h, but decreased rapidly from 70 to 55 pg/ml thereafter. Similarly, the TNF-α levels increased slowly from 80 to 90 pg/ml during the first 8 h and rapidly from 90 to 145 pg/ml in subsequent hours with the silence of miR-330-3p (Fig. 3f). Collectively, these results suggest that TNF-α is a direct target of miR-330-3p, and that the endogenous TNF-α is tightly regulated by miR-330-3p in synoviocytes during OA pathogenesis.

### CircGCN1L1 induces synoviocyte proliferation, chondrocyte apoptosis, and cartilage ECM degradation

**Fig. 4:**
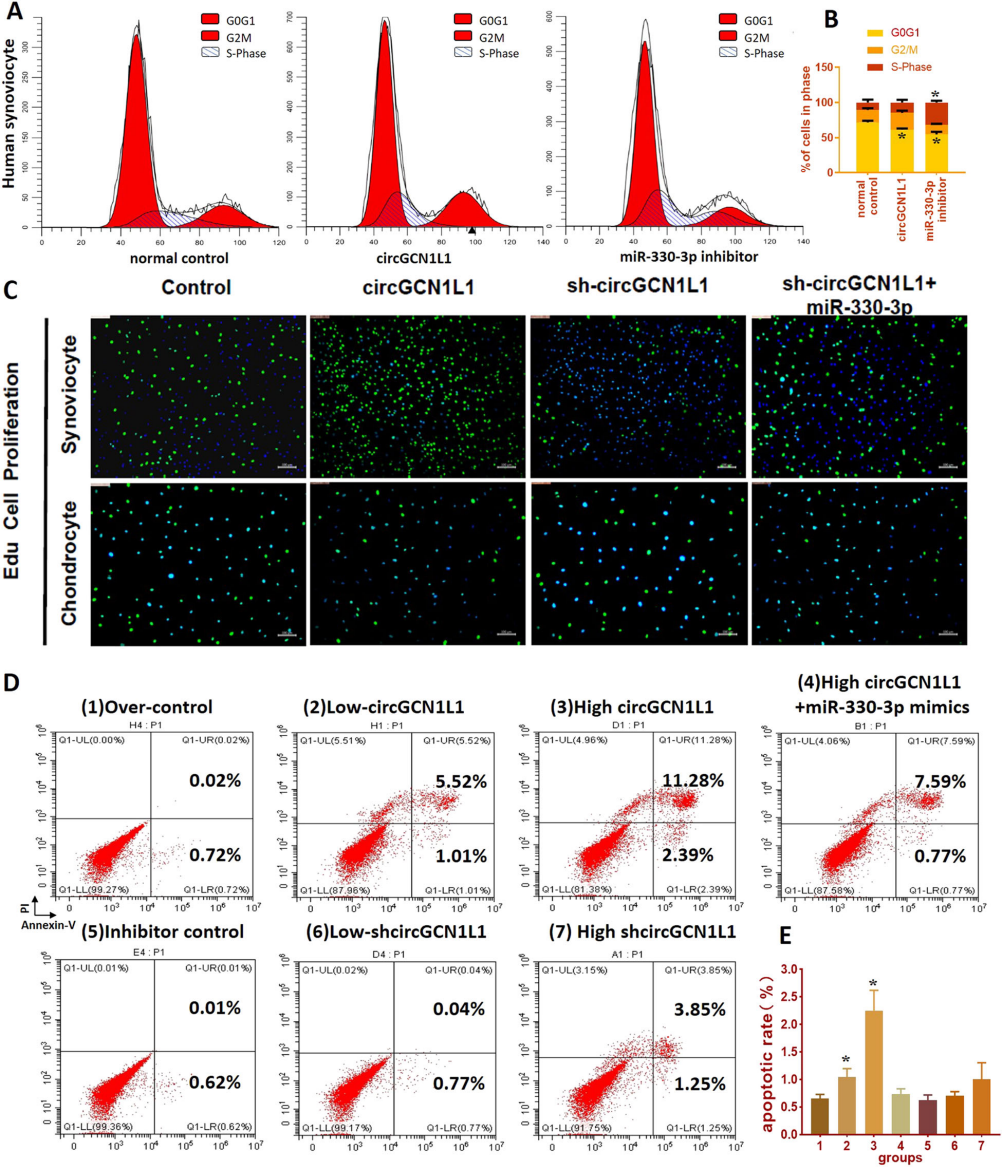
CircGCN1L1 interacts with miR-330-3p and is involved in the regulation of synoviocyte proliferation and chondrocyte apoptosis. **a**, **b** Human synoviocytes from the control patient were transfected with circGCN1L1 plasmid or miR-330-3p inhibitor. The proportion of cells in the G1, S, or G2 phase was detected using quantitative FACS analysis. *N* = 6 (two different samples for three independent experiments). *p < 0.05. **c** Edu cell proliferation assay on single cultured synoviocytes from the control patient and co-cultured chondrocyte. Synoviocytes were divided into different groups according to the transfection (circGCN1L1, circGCN1L1 control, shcircGCN1L1, shcircGCN1L1 control, shcircGCN1L1 and miR-330-3p, circGCN1L1 and miR-330-3p inhibitor, and blank control). Edu-labeled proliferating cells (green), DAPI-labeled nuclei (blue). *N* = 3 (one sample for three independent experiments). Scale bar, 500 μm. **d** TMJ synoviocytes from the control patient were transfected with one of various concentrations of plasmids and then co-cultured with condylar chondrocytes. Chondrocyte apoptosis was detected with Annexin V-FITC/PI double staining using quantitative FACS analysis. *p < 0.05. Data are presented as mean ± S.D. One-way ANOVA with Bonferroni test (**c**–**f**) was performed. FACS f﻿luorescence-activated cell sorting, PI propidium iodide, FITC fluorescein isothiocyanate, DAPI dihydrochloride, Edu 5-ethynyl-2′-deoxyuridine, TMJ temporomandibular joint.

**Fig. 5:**
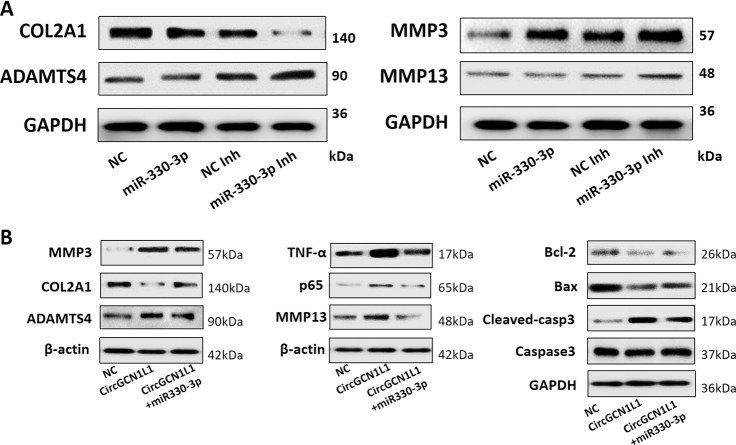
MiR-330-3p mediates the function of circGCN1L1 in human TMJ synoviocyte. **a** The synovial cells from the control patient were transiently transfected with miR-330-3p mimics, miR-330-3p mimic NC, miR-330-3p inhibitor, or miR-330-3p inhibitor NC, respectively. Forty-eight hours later, the levels of MMP13, MMP3, COL2A1, and ADAMTS4 were detected using WB. *N* = 4 (four independent experiments). **b** CircGCNL1 with/without miR-330-3p mimics was transfected into co-cultured synoviocytes. After 2 days of co-culture, proteins were extracted from chondrocytes in the lower chamber, and WB was performed to detect the levels of MMP13, MMP3, COL2A1, ADAMTS4, TNF-α, p65, Bcl-2, Bax, caspase-3, and cleaved caspase-3. *N* = 4 (four independent experiments). Data are presented as mean ± S.D. One-way ANOVA with Bonferroni test was performed. WB western blotting, NC normal control, MMP matrix metalloproteinase, TNF tumor necrosis factor, Bcl-2 B-cell lymphoma-2, Bax BCL2-associated X, COL2A1 collagen type II alpha 1.

Clinical observations and researches indicate that the synovial membrane in patients with OA often displays proliferation and thickening in response to chronic inflammation^30,31^. Therefore, we added circGCN1L1 mimics and miRNA 330-3p inhibitor plasmids to primary synovial cells and then detected changes using propidium iodide (PI) staining and flow cytometry. In the group transfected with circGCN1L1 mimics, the number of synovial cells in the G0 phase decreased, whereas the number of cells in the S phase increased, suggesting that circGCN1L1 overexpression increased the number of synovial cells entering the proliferative phase (Fig. 4a, b). The GSEA revealed the enrichment of the differentially expressed genes identified in TMJOA synovium in the hallmark gene set of apoptosis and TNF-α signaling via NF-κB (Supplementary Fig. 4). Therefore, a co-culture system based on the Transwell chamber was established to determine the effect of circGCN1L1 on the phenotypes of TMJ synoviocytes and condylar chondrocytes. The Edu cell proliferation experiments revealed an increase in synoviocyte proliferation following circGCN1L1 overexpression, whereas the silencing of circGCN1L1 inhibited cell proliferation. The simultaneous transfection of miR-330-3p mimics offsets the effect of circGCN1L1 inhibition on synoviocytes. When the transfected synoviocyte was co-cultured with chondrocytes, the circGCN1L1-overexpressing synoviocytes significantly inhibited chondrocyte proliferation, whereas the circGCN1L1-silenced synoviocytes exerted little effect on chondrocyte proliferation (Fig. 4c). We further used the co-culture system to determine whether synovial circGCN1L1 modulates chondrocyte apoptosis. As shown in Fig. 4d and e, the overexpression of circGCN1L1 in synoviocytes significantly increased the percentage of apoptotic condylar chondrocytes (Fig. 4d1–3), whereas the percentage of these cells was markedly decreased by the miR-330-3p sponge (Fig. 4d1, 4). However, the inhibition of circGCN1L1 in synoviocytes exerted little effect on chondrocyte apoptosis (Fig. 4d5–7). In addition, when circGCNL1 was overexpressed at lower levels in synoviocytes, the chondrocytes affected were mainly in the early cell apoptosis stage (Fig. 4d1, 2). However, as circGCN1L1 overexpression increased, the affected chondrocytes were mainly undergoing late apoptosis or necrosis (Fig. 4d1, 3). Subsequently, we synthesized miR-330-3p analogs, including miR-330-3p mimics, miR-330-3p mimic NC, miR-330-3p inhibitor, and miR-330-3p inhibitor NC, and transiently transfected these analogs into human synoviocytes. The results of western blot show that COL2A1 was significantly decreased, whereas MMP13, MMP3, and ADAMTS4 levels were conversely increased after the downregulation of miR-330-3p, suggesting that miR-330-3p knockdown increased the secretion of matrix-degrading enzymes in synovial cells (Fig. 5a, Supplementary Fig. 8). Moreover, circGCNL1 with/without miR-330-3p mimics was transfected into co-cultured synoviocytes, and proteins were extracted from chondrocytes in the lower chamber. The level of COL2A1 in chondrocytes decreased after the upregulation of circGCNL1, whereas the levels of cell matrix-degrading enzymes, such as MMP13, MMP3, and ADAMTS4, had increased significantly. Apoptotic factors associated with NF-κB signaling, such as TNF-α, p65, Bcl-2, Bax, and activated caspase-3, showed the same trends. Those functional experiments suggested that circGCNL1 contributes to chondrocyte ECM degeneration (Fig. 5b, Supplementary Fig. 9). These data suggested that circGCNL1 upregulation in synoviocytes could trigger activated catabolism and conversely attenuated anabolism together with an inflammatory cascade, which continuously leads to chondrocyte apoptosis. Those effects were conversely downregulated by the miR-330-3p sponge in human synoviocyte at both the mRNA (Fig. 2e) and protein levels, which indicated that circGCN1L1 may potentially function as a sponge and targets miR-330-3p to modulate TNF-α expression in human TMJ synoviocytes.

### CircGCN1L1 downregulation alleviates joint degeneration in the TMJOA rat model

**Fig. 6:**
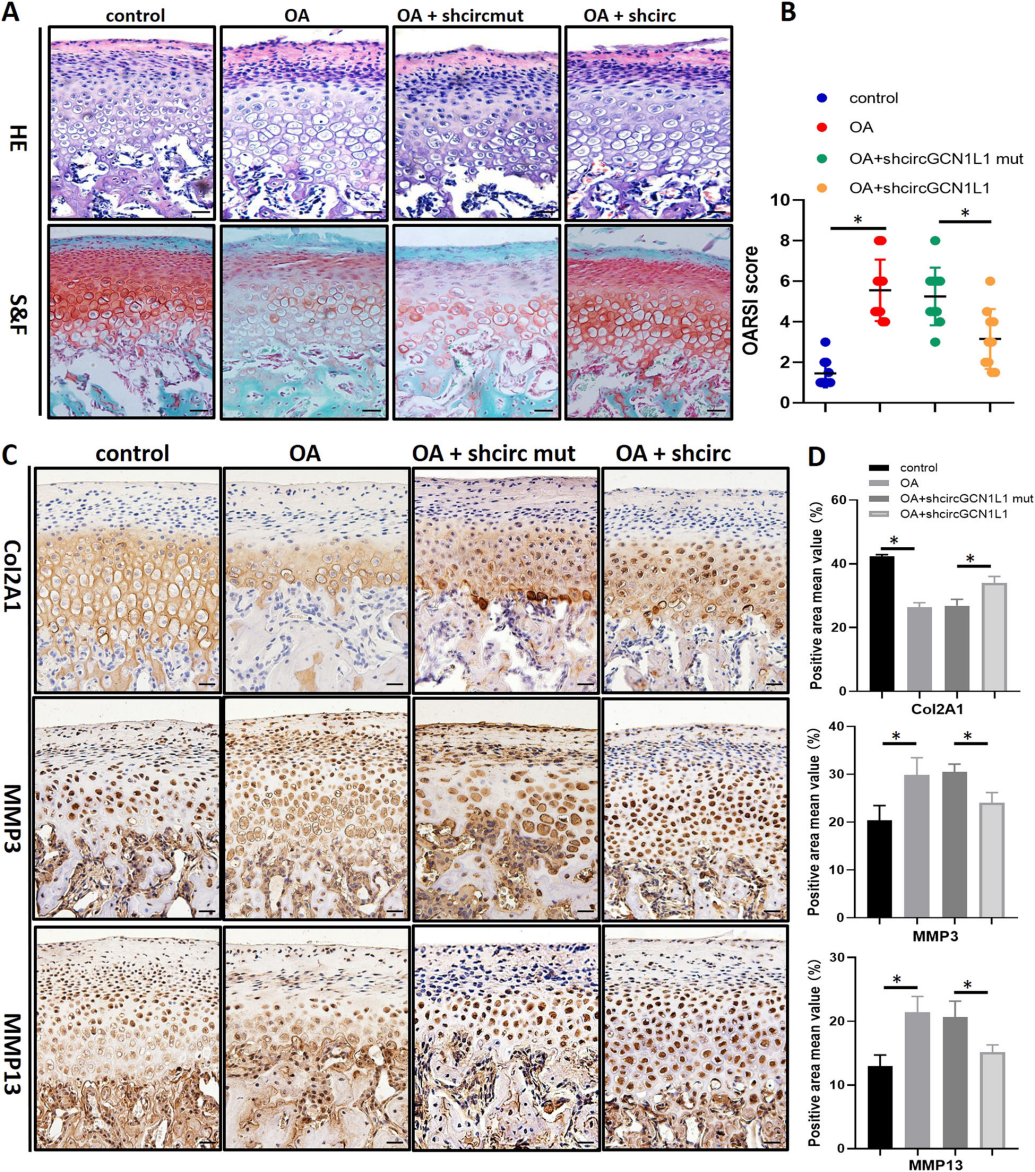
Inhibiting the expression of circGCN1L1 in a TMJOA rat model slows the progression of osteoarthritis. **a**, **b** HE and Safranin-O/Fast green staining of the cartilage in control and occlusion-induced OA rats with shcircGCN1L or shcircGCN1L1 MT injected. Scale bar, 50 μm. OARSI scoring was performed according to the staining results; *N* = 40 (10 rats in each group). *p < 0.05. **c**, **d** IHC staining in control and occlusion-induced OA rats with shcircGCN1L or shcircGCN1L1 MT injected. Scale bar, 50 μm. *N* = 40 (10 rats in each group). *p < 0.05. Data are presented as mean ± S.D. One-way ANOVA with Bonferroni test was performed. HE hematoxylin–eosin, IHC immunohistochemistry, MT mutant, OA osteoarthritis, OARSI Osteoarthritis Research Society International.

**Fig. 7:**
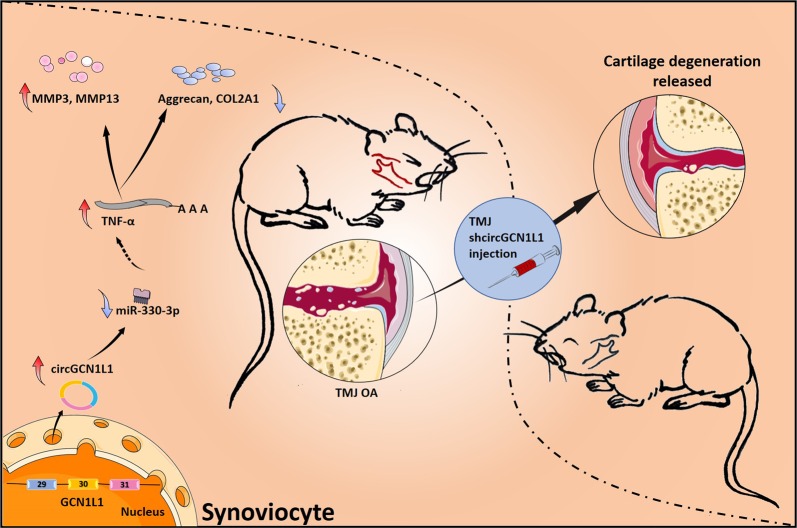
Schematic of the working hypothesis. Novel circGCN1L1 in synoviocyte may play a role as miR-330-3p sponge to upregulate TNF-α expression in TMJOA and alleviate TMJOA in vivo.

Since the occlusion disorder can produce abnormalities in bite force direction and distribution, which resemble the pathogenesis of human OA, occlusal-induced OA is regarded as a suitable model to analyze pathogenesis. To investigate the functions of circGCN1L1 in vivo, shcircGCN1L1, shcircGCN1L1 MT, or PBS were intra-articularly injected into occlusal-induced TMJOA separately. After 1 month, HE and Safranin-O/Fast green staining were performed to analyze the results. Compared with the control group, the condylar fiber layer in the TMJOA group was thickened and displayed an empty cartilage lacuna. The chondrocyte cluster formed a disordered layer with a loose distribution, a calcified cartilage layer, and subchondral bone formation disorders. Cartilage degradation in occlusal-induced TMJOA rats was decreased by shcircGCN1L1 injection but not by shcircGCN1L1 MT injection. Quantitative analysis indicated that shcircGCN1L1-injected rats demonstrated significantly lower OARSI scores, while shcircGCN1L1 MT-injected rats had higher scores (Fig. 6a, b). Moreover, the IHC showed that shcircGCN1L1, but not shcircGCN1L1 MT, reversed COL2A1 loss and upregulated matrix catabolic enzymes such as MMP3 and MMP13 (Fig. 6c, d, Supplementary Table 1S5). Together, these results revealed the positive effects of decreased circGCN1L1 expression that could inhibit ECM catabolism and alleviate TMJOA in vivo (Fig. 7).

## Discussion

Drugs approved for the clinical treatment of TMJOA have limited effects. The underlying mechanisms of TMJOA must be elucidated. Aseptic inflammation in the TMJ synovium (synovitis) is the early manifestation of OA and is the main source of TMJ pain^32,33^. Once synovitis can be blocked, the inflammatory cascade throughout the joint can be prevented, thereby attenuating the inflammatory response, alleviating pain, and stopping the development and aggravation of TMJOA^34,35^. Circular RNAs are a type of endogenous noncoding RNA that performs very important roles in OA progression^8,36^. Several studies have reported differential expression of circRNAs in OA cartilage and their potential to regulate the vitality and functions of chondrocytes^11,37^. However, little is known about the roles of circRNAs in the articular synovium. No studies of circRNAs in TMJOA have been reported to date. Therefore, an analysis of the cellular mechanisms of synovial circRNAs in TMJOA may provide new perspectives for early OA control and treatment.

In our study, circRNAs were differentially expressed in the TMJOA synovium, including a novel upregulated circRNA, circGCN1L1, that is involved in TMJ synovitis^18^. Moreover, we elucidated the mechanism by which synovial circGCN1L1 regulates the expression of a key inflammatory cytokine, TNF-α, and its effects on the phenotypes of synoviocytes and TMJ condylar chondrocytes using multiple strategies in vitro and in vivo. We identified a new circRNA involved in TMJ synovitis progression that represents a promising candidate for early TMJOA treatment. CircGCN1L1 is generated by the back-splicing of exons 29–31 of the GCN1L1 gene. The circGCN1L1 gene sequence (750 bp) shares 87% similarity to the rat genome alignment. Furthermore, the human and rat circGCN1L1 (389 bp) sequence displays up to 90% similarity. In addition, miR-330-3p, TNF-α, and their binding site are conserved in rats. Hence, a rat TMJOA model was used for the in vivo validation of the effects of circGCN1L1 inhibition. In our study, both GCN1L1 and circGCN1L1 were upregulated in patients with TMJOA relative to control patients. GCN1L1 functions as a positive activator of EIF2AK4/GCN2 protein kinase activity in response to amino acid starvation. An independent role for circGCN1L1 in TMJOA was established in the present study.

CeRNA networks have been validated to involve various noncoding RNAs (ncRNAs) and to play roles in many diseases, including OA^10^. CircRNAs possess a circular structure and tissue-specific expression that differs from that of linear ncRNAs, and they have attracted increasing attention in recent years^26,38,39^. Because circRNAs contain abundant microRNA-binding sites, they may function as miRNA sponges to indirectly regulate the functions of target miRNAs. Several circRNA–miRNA–target gene axes have been confirmed to participate in the initiation and development of many diseases. For example, the circSERPINE2–miR-1271–ERG axis in knee cartilage has been identified as a novel target for the prevention and treatment of knee OA^37^. However, researchers have not determined whether circRNAs are involved in the early TMJOA stage (synovitis). In the present study, synovial circGCN1L1 containing a miR-330-3p target site was identified using RNA seq and validated using gain-of-function and loss-of-function strategies, FISH, and luciferase report assays. Furthermore, miR-330-3p was found to target TNF-α and to be regulated by circGCN1L1. Therefore, we propose that circGCN1L1 serves as a miR-330-3p sponge to promote the expression of a key inflammatory cytokine in TMJOA through ceRNA mechanism. Our in vitro and in vivo experiments also confirmed that circGCN1L1 promotes synoviocyte proliferation and chondrocyte apoptosis through miR-330-3p and TNF-α. The high level of TNF-α in the microenvironment can be expected to accelerate disease progression. However, we are unable to exclude the possible involvement of other critical circRNAs, miRNAs, and target genes in addition to circGCN1L1, miR-330-3p, and TNF that may play important roles in the progression of OA.

To clarify the importance of circGCN1L1 in the TMJOA process, we established a model of unilateral anterior teeth recombination to induce the occurrence of TMJOA in SD rats. Relative to the NC group, the TMJOA model group displayed a loss of the cartilage matrix, rough articular cartilage surface fibers, and thinning or loss of the proliferative zone, and some cartilage layers were obviously thickened. In addition, undifferentiated mesenchymal cells in the proliferative zone and chondrocytes in the hypertrophic zone were much more than that in the control group. Furthermore, the dense bone was thinner, and vacuoles had formed in the TMJOA group. The histopathological diagnosis confirmed the stable and effective establishment of the OA model. Under this circumstance, we injected shcircGCN1L1 MT plasmid into the joint cavity and found that the TMJOA phenotype was not significantly alleviated, whereas in the shcircGCN1L1-injected group, proteoglycan loss was reduced in the cartilage. Immunohistochemical staining revealed increased expression of COL2Al in these rats relative to that in the occlusion-induced group, whereas the expression of MMP3 and MMP13 was contrarily decreased. The in vivo experiments indicate that the inhibition of circGCN1L1 in the joint cavity slows the progression of TMJ inflammation and reduces the expression of matrix-degrading enzymes, thereby decreasing the loss of condylar cartilage and subchondral bone.

In conclusion, our research identified that the circGCN1L1–miR-330-3p–TNF axis may serve as a new target for the early prevention and treatment of TMJOA. Repression of circGCN1L1 may reduce synovial hyperplasia and chondrocyte apoptosis by increasing ECM formation. In addition, targeting miR-330-3p may represent another potential treatment option.

## Supplementary information

Supplementary Figure legends

Supplementary Table 1

Supplementary Table 2

Supplementary Table 3

Supplementary Figure 1

Supplementary Figure 2

Supplementary Figure 3

Supplementary Figure 4

Supplementary Figure 5

Supplementary Figure 6

Supplementary Figure 7

Supplementary Figure 8

Supplementary Figure 9
