# Supplementary material for: CircGCN1L1 promotes synoviocyte proliferation and chondrocyte apoptosis by targeting miR-330-3p and TNF-α in TMJ osteoarthritis
Source: Cell Death Dis. 2020 Apr 24;11(4):284. doi: 10.1038/s41419-020-2447-7 (PMC7181816; doi:10.1038/s41419-020-2447-7)
Supplement: Supplementary file 1 — Supplementary Figure legends [file 41419_2020_2447_MOESM1_ESM.docx]

Supplementary Figure 1. Overexpression plasmid vector for circGCN1L1 (GPLVX-Laccase2-hsa_circ_0Y000448-Puro).

Supplementary Figure 2. ShRNA vector sequence (hsa_circ_0000448 shRNA (PGMLV-SC5)).

Supplementary Figure 3. Overexpression vector for circGCN1L1 and human TNF UTR in Luciferase assay.

Supplementary Figure 4. Gene set enrichment analysis of the differentially expressed genes identified in TMJOA synovium. P<0.05.

Supplementary Figure 5. Prediction of circRNA-miRNA interactions using circRNA-Interactome, StarBase, and RegRNA2.0 softwares.

Supplementary Figure 6. The knockdown efficiency and the expression of GCN1L1 were evaluated by RT-qPCR. Data are presented as mean ± S.D. Two tailed t-test was performed.

Supplementary Figure 7. Prediction of mRNA-miRNA interactions using TargetScan, StarBase, and miRanda softwares.

Supplementary Figure 8. Densitometric quantification of western blot results in Figure 5A. Data are presented as mean ± S.D. One-way ANOVA with Bonferroni test was performed.

Supplementary Figure 9. Densitometric quantification of western blot results in Figure 5B. Data are presented as mean ± S.D. One-way ANOVA with Bonferroni test was performed.

Supplementary Table 1. Sample information and OARSI scores.

Supplementary Table 2. Primers and sequences used in this study.

Supplementary Table 3. Differentially expressed circRNAs between TMJOA and control synovial samples.
