# Supplementary material for: CircGCN1L1 promotes synoviocyte proliferation and chondrocyte apoptosis by targeting miR-330-3p and TNF-α in TMJ osteoarthritis
Source: Cell Death Dis. 2020 Apr 24;11(4):284. doi: 10.1038/s41419-020-2447-7 (PMC7181816; doi:10.1038/s41419-020-2447-7)
Supplement: Supplementary file 2 — Supplementary Table 1 [file 41419_2020_2447_MOESM2_ESM.docx]

**Supplementary Table 1. Sample information and OARSI scores.**

| **S1. Baseline data of enrolled patients.** | | | | | | | | | | | | | |
| --- | --- | --- | --- | --- | --- | --- | --- | --- | --- | --- | --- | --- | --- |
| ***No.*** | | ***Name*** | | ***Sex*** | | ***Age*** | | ***Diagnosis*** | | ***Clinical Stage*** | | ***Group*** | |
| ***01*** | | ***ZZY*** | | ***Female*** | | ***28 y*** | | ***TMJOA*** | | ***Wilkes Stage 4*** | | ***Experiment*** | |
| ***02*** | | ***XSX*** | | ***Female*** | | ***37 y*** | | ***TMJOA*** | | ***Wilkes Stage 4*** | | ***Experiment*** | |
| ***03*** | | ***WSY*** | | ***Female*** | | ***41 y*** | | ***TMJOA*** | | ***Wilkes Stage 4*** | | ***Experiment*** | |
| ***04*** | | ***WL*** | | ***Female*** | | ***38 y*** | | ***TMJOA*** | | ***Wilkes Stage 4*** | | ***Experiment*** | |
| ***05*** | | ***ZYJ*** | | ***Female*** | | ***42 y*** | | ***TMJOA*** | | ***Wilkes Stage 4*** | | ***Experiment*** | |
| ***06*** | | ***SF*** | | ***Female*** | | ***37 y*** | | ***TMJOA*** | | ***Wilkes Stage 4*** | | ***Experiment*** | |
| ***07*** | | ***FRR*** | | ***Female*** | | ***31 y*** | | ***TMJOA*** | | ***Wilkes Stage 4*** | | ***Experiment*** | |
| ***08*** | | ***DWF*** | | ***Female*** | | ***34 y*** | | ***TMJOA*** | | ***Wilkes Stage 4*** | | ***Experiment*** | |
| ***09*** | | ***ZXL*** | | ***Female*** | | ***40 y*** | | ***TMJOA*** | | ***Wilkes Stage 4*** | | ***Experiment*** | |
| ***10*** | | ***QXL*** | | ***Female*** | | ***36 y*** | | ***TMJOA*** | | ***Wilkes Stage 4*** | | ***Experiment*** | |
| ***11*** | | ***HYY*** | | ***Female*** | | ***32 y*** | | ***TMJOA*** | | ***Wilkes Stage 4*** | | ***Experiment*** | |
| ***12*** | | ***WFR*** | | ***Female*** | | ***41 y*** | | ***TMJOA*** | | ***Wilkes Stage 4*** | | ***Experiment*** | |
| ***13*** | | ***GX*** | | ***Female*** | | ***35 y*** | | ***TMJOA*** | | ***Wilkes Stage 4*** | | ***Experiment*** | |
| ***14*** | | ***FR*** | | ***Female*** | | ***37 y*** | | ***TMJOA*** | | ***Wilkes Stage 4*** | | ***Experiment*** | |
| ***15*** | | ***OL*** | | ***Female*** | | ***34 y*** | | ***TMJOA*** | | ***Wilkes Stage 4*** | | ***Experiment*** | |
| ***16*** | | ***QWY*** | | ***Female*** | | ***31 y*** | | ***TMJOA*** | | ***Wilkes Stage 4*** | | ***Experiment*** | |
| ***17*** | | ***WRQ*** | | ***Female*** | | ***33 y*** | | ***TMJOA*** | | ***Wilkes Stage 4*** | | ***Experiment*** | |
| ***18*** | | ***YJM*** | | ***Female*** | | ***41 y*** | | ***TMJOA*** | | ***Wilkes Stage 4*** | | ***Experiment*** | |
| ***19*** | | ***MZH*** | | ***Female*** | | ***29 y*** | | ***TMJOA*** | | ***Wilkes Stage 4*** | | ***Experiment*** | |
| ***20*** | | ***HZR*** | | ***Female*** | | ***39 y*** | | ***TMJOA*** | | ***Wilkes Stage 4*** | | ***Experiment*** | |
| ***21*** | | ***LR*** | | ***Female*** | | ***34 y*** | | ***TMD*** | | ***Wilkes Stage 2-3*** | | ***Control*** | |
| ***22*** | | ***ZYC*** | | ***Female*** | | ***39 y*** | | ***TMD*** | | ***Wilkes Stage 2-3*** | | ***Control*** | |
| ***23*** | | ***YXY*** | | ***Female*** | | ***35 y*** | | ***TMD*** | | ***Wilkes Stage 2-3*** | | ***Control*** | |
| ***24*** | | ***JQ*** | | ***Female*** | | ***40 y*** | | ***TMD*** | | ***Wilkes Stage 2-3*** | | ***Control*** | |
| ***25*** | | ***JM*** | | ***Female*** | | ***46 y*** | | ***TMD*** | | ***Wilkes Stage 2-3*** | | ***Control*** | |
| ***26*** | | ***NXC*** | | ***Female*** | | ***34 y*** | | ***TMD*** | | ***Wilkes Stage 2-3*** | | ***Control*** | |
| ***27*** | | ***LSA*** | | ***Female*** | | ***35 y*** | | ***TMD*** | | ***Wilkes Stage 2-3*** | | ***Control*** | |
| ***28*** | | ***WJF*** | | ***Female*** | | ***47 y*** | | ***TMD*** | | ***Wilkes Stage 2-3*** | | ***Control*** | |
| ***29*** | | ***WYH*** | | ***Female*** | | ***36 y*** | | ***TMD*** | | ***Wilkes Stage 2-3*** | | ***Control*** | |
| ***30*** | | ***XZP*** | | ***Female*** | | ***39 y*** | | ***TMD*** | | ***Wilkes Stage 2-3*** | | ***Control*** | |
| ***31*** | | ***DSE*** | | ***Female*** | | ***33 y*** | | ***TMD*** | | ***Wilkes Stage 2-3*** | | ***Control*** | |
| ***32*** | | ***DJ*** | | ***Female*** | | ***42 y*** | | ***TMD*** | | ***Wilkes Stage 2-3*** | | ***Control*** | |
| ***33*** | | ***MK*** | | ***Female*** | | ***41 y*** | | ***TMD*** | | ***Wilkes Stage 2-3*** | | ***Control*** | |
| ***34*** | | ***SY*** | | ***Female*** | | ***37 y*** | | ***TMD*** | | ***Wilkes Stage 2-3*** | | ***Control*** | |
| ***35*** | | ***SLX*** | | ***Female*** | | ***36 y*** | | ***TMD*** | | ***Wilkes Stage 2-3*** | | ***Control*** | |
| ***36*** | | ***ZHM*** | | ***Female*** | | ***36 y*** | | ***TMD*** | | ***Wilkes Stage 2-3*** | | ***Control*** | |
| ***37*** | | ***YJ*** | | ***Female*** | | ***45 y*** | | ***TMD*** | | ***Wilkes Stage 2-3*** | | ***Control*** | |
| ***38*** | | ***SJW*** | | ***Female*** | | ***39 y*** | | ***TMD*** | | ***Wilkes Stage 2-3*** | | ***Control*** | |
| ***39*** | | ***CXX*** | | ***Female*** | | ***43 y*** | | ***TMD*** | | ***Wilkes Stage 2-3*** | | ***Control*** | |
| ***40*** | | ***QJY*** | | ***Female*** | | ***40 y*** | | ***TMD*** | | ***Wilkes Stage 2-3*** | | ***Control*** | |
| **Abbreviations: TMJOA (temporomandibular osteoarthritis), TMD (temporo- mandibular disorders).** | | | | | | | | | | | | | |
| **S2. Clinical symptoms of enrolled patients.** | | | | | | | | | | | | |  |
| ***No.*** | ***Name*** | | ***Pain*** | | ***Clicking*** | | ***MIO*** | | ***Hydrarthrosis*** | | ***Articular Disc & Bone Changes*** | |  |
| ***01*** | ***ZZY*** | |  | |  | | ***25 mm*** | | ***√*** | | ***R-ADDWo/R with bony changes*** | |  |
| ***02*** | ***XSX*** | |  | |  | | ***26 mm*** | | ***√*** | | ***Bi-ADDWo/R with bony changes*** | |  |
| ***03*** | ***WSY*** | |  | |  | | ***20 mm*** | | ***√*** | | ***L-ADDWo/R with bony changes*** | |  |
| ***04*** | ***WL*** | |  | |  | | ***24 mm*** | | ***√*** | | ***Bi-ADDWo/R with bony changes*** | |  |
| ***05*** | ***ZYJ*** | |  | |  | | ***17 mm*** | | ***√*** | | ***R-ADDWo/R with bony changes*** | |  |
| ***06*** | ***SF*** | |  | | ***√*** | | ***19 mm*** | | ***√*** | | ***L-ADDWo/R with bony changes*** | |  |
| ***07*** | ***FRR*** | | ***√*** | |  | | ***21 mm*** | | ***√*** | | ***L-ADDWo/R with bony changes*** | |  |
| ***08*** | ***DWF*** | |  | |  | | ***20 mm*** | | ***√*** | | ***Bi-ADDWo/R with bony changes*** | |  |
| ***09*** | ***ZXL*** | | ***√*** | |  | | ***26 mm*** | | ***√*** | | ***R-ADDWo/R with bony changes*** | |  |
| ***10*** | ***QXL*** | | ***√*** | |  | | ***22 mm*** | | ***√*** | | ***R-ADDWo/R with bony changes*** | |  |
| ***11*** | ***HYY*** | |  | |  | | ***18 mm*** | | ***√*** | | ***L-ADDWo/R with bony changes*** | |  |
| ***12*** | ***WFR*** | |  | |  | | ***22 mm*** | | ***√*** | | ***Bi-ADDWo/R with bony changes*** | |  |
| ***13*** | ***GX*** | |  | |  | | ***24 mm*** | | ***√*** | | ***R-ADDWo/R with bony changes*** | |  |
| ***14*** | ***FR*** | |  | | ***√*** | | ***20 mm*** | | ***√*** | | ***R-ADDWo/R with bony changes*** | |  |
| ***15*** | ***OL*** | |  | |  | | ***21 mm*** | | ***√*** | | ***R-ADDWo/R with bony changes*** | |  |
| ***16*** | ***QWY*** | |  | |  | | ***23 mm*** | | ***√*** | | ***L-ADDWo/R with bony changes*** | |  |
| ***17*** | ***WRQ*** | | ***√*** | |  | | ***19 mm*** | | ***√*** | | ***Bi-ADDWo/R with bony changes*** | |  |
| ***18*** | ***YJM*** | |  | |  | | ***26 mm*** | | ***√*** | | ***R-ADDWo/R with bony changes*** | |  |
| ***19*** | ***MZH*** | |  | | ***√*** | | ***22 mm*** | | ***√*** | | ***R-ADDWo/R with bony changes*** | |  |
| ***20*** | ***HZR*** | |  | |  | | ***18 mm*** | | ***√*** | | ***L-ADDWo/R with bony changes*** | |  |
| ***21*** | ***LR*** | | ***none*** | | ***√*** | | ***30 mm*** | | ***none*** | | ***Bi-ADDWo/R without bony changes*** | |  |
| ***22*** | ***ZYC*** | | ***none*** | | ***√*** | | ***31 mm*** | | ***none*** | | ***L-ADDWo/R without bony changes*** | |  |
| ***23*** | ***YXY*** | | ***none*** | | ***√*** | | ***27 mm*** | | ***none*** | | ***Bi-ADDWo/R without bony changes*** | |  |
| ***24*** | ***JQ*** | | ***none*** | | ***√*** | | ***32 mm*** | | ***none*** | | ***Bi-ADDWo/R without bony changes*** | |  |
| ***25*** | ***JM*** | | ***none*** | | ***√*** | | ***31 mm*** | | ***none*** | | ***L-ADDWo/R without bony changes*** | |  |
| ***26*** | ***NXC*** | | ***none*** | | ***√*** | | ***27 mm*** | | ***none*** | | ***R-ADDWo/R without bony changes*** | |  |
| ***27*** | ***LSA*** | | ***none*** | | ***√*** | | ***32 mm*** | | ***none*** | | ***R-ADDWo/R without bony changes*** | |  |
| ***28*** | ***WJF*** | | ***none*** | | ***√*** | | ***30 mm*** | | ***none*** | | ***L-ADDWo/R without bony changes*** | |  |
| ***29*** | ***WYH*** | | ***none*** | | ***√*** | | ***29 mm*** | | ***none*** | | ***Bi-ADDWo/R without bony changes*** | |  |
| ***30*** | ***XZP*** | | ***none*** | | ***√*** | | ***28 mm*** | | ***none*** | | ***R-ADDWo/R without bony changes*** | |  |
| ***31*** | ***DSE*** | | ***none*** | | ***√*** | | ***28 mm*** | | ***none*** | | ***Bi-ADDWo/R without bony changes*** | |  |
| ***32*** | ***DJ*** | | ***none*** | | ***√*** | | ***27 mm*** | | ***none*** | | ***Bi-ADDWo/R without bony changes*** | |  |
| ***33*** | ***MK*** | | ***none*** | | ***√*** | | ***33 mm*** | | ***none*** | | ***L-ADDWo/R without bony changes*** | |  |
| ***34*** | ***SY*** | | ***none*** | | ***√*** | | ***32 mm*** | | ***none*** | | ***R-ADDWo/R without bony changes*** | |  |
| ***35*** | ***SLX*** | | ***none*** | | ***√*** | | ***33 mm*** | | ***none*** | | ***R-ADDWo/R without bony changes*** | |  |
| ***36*** | ***ZHM*** | | ***none*** | | ***√*** | | ***32 mm*** | | ***none*** | | ***Bi-ADDWo/R without bony changes*** | |  |
| ***37*** | ***YJ*** | | ***none*** | | ***√*** | | ***31 mm*** | | ***none*** | | ***R-ADDWo/R without bony changes*** | |  |
| ***38*** | ***SJW*** | | ***none*** | | ***√*** | | ***29 mm*** | | ***none*** | | ***Bi-ADDWo/R without bony changes*** | |  |
| ***39*** | ***CXX*** | | ***none*** | | ***√*** | | ***29 mm*** | | ***none*** | | ***Bi-ADDWo/R without bony changes*** | |  |
| ***40*** | ***QJY*** | | ***none*** | | ***√*** | | ***30 mm*** | | ***none*** | | ***L-ADDWo/R without bony changes*** | |  |
| ***Abbreviations: Bi (bilateral), R (right), L (left), MIO (maximal interincisal opening), ADDWo/R (anterior disc displacement without reduction), “√” = having related clinical symptoms.*** | | | | | | | | | | | | |  |

**S3. Patients with TMJ condylar fracture (N=3).**

| ***No.*** | ***Name*** | ***Sex*** | ***Age (y)*** | ***Diagnosis*** |
| --- | --- | --- | --- | --- |
| ***01*** | **CHQ** | **Male** | **28** | **Bilateral condylar fracture** |
| ***02*** | **YZY** | **Female** | **22** | **Bilateral condylar fracture** |
| ***03*** | **ZYN** | **Female** | **42** | **Left condylar fracture** |

**S4. Top 10 differentially-expressed CircRNAs for validation.**

| ***No.*** | ***CircRNAID*** | ***circBase ID*** | ***P-value*** | ***Fold Change*** | ***Regulation*** |
| --- | --- | --- | --- | --- | --- |
| ***01*** | **chr15:59204762-59209198-** | **hsa_circ_0000605** | **0.004** | **162.17** | **Up** |
| ***02*** | **chr12:120592774-120593523-** | **hsa_circ_0000448** | **0.005** | **153.30** | **Up** |
| ***03*** | **chr21:37711073-37717005+** | **none** | **0.005** | **104.71** | **Up** |
| ***04*** | **chr2:72945232-72960247-** | **hsa_circ_0009043** | **0.001** | **425.31** | **Down** |
| ***05*** | **chr5:134076753-134079742+** | **hsa_circ_0003154** | **0.043** | **71.01** | **Down** |
| ***06*** | **chr12:12397196-12397589-** | **hsa_circ_0000378** | **0.035** | **62.68** | **Down** |
| ***07*** | **chr5:95091100-95099324+** | **hsa_circ_0007444** | **0.014** | **55.09** | **Down** |
| ***08*** | **chr17:33495080-33495704+** | **none** | **0.039** | **47.18** | **Down** |
| ***09*** | **chr8:52773405-52773806-** | **hsa_circ_0001801** | **0.030** | **46.20** | **Down** |
| ***10*** | **chrX:139865340-139866824+** | **hsa_circ_0001946** | **0.009** | **2.73** | **Down** |

**S5.** **Details of OARSI scoring**

| ***Groups*** | ***avg*** | ***SD*** | ***max*** | ***min*** |
| --- | --- | --- | --- | --- |
| ***control*** | **1.45** | **0.69** | **3** | **1** |
| ***OA*** | **5.55** | **1.52** | **8** | **4** |
| ***OA + shcirc mut*** | **5.25** | **1.42** | **8** | **3** |
| ***OA + shcirc*** | **3.15** | **1.47** | **6** | **1.5** |

***The significance between control and OA is 0.000, between OA and OA + shcircGCN1L1 mut is 0.604, between OA + shcircGCN1L1 and OA + shcircGCN1L1 mut is 0.022. Abbreviations:* avg (average), SD (Standard Deviation), max (maximum), min (minimum).**
