# Supplementary material for: CircGCN1L1 promotes synoviocyte proliferation and chondrocyte apoptosis by targeting miR-330-3p and TNF-α in TMJ osteoarthritis
Source: Cell Death Dis. 2020 Apr 24;11(4):284. doi: 10.1038/s41419-020-2447-7 (PMC7181816; doi:10.1038/s41419-020-2447-7)
Supplement: Supplementary file 3 — Supplementary Table 2 [file 41419_2020_2447_MOESM3_ESM.docx]

**Supplementary Table 2. Primers and sequences used in this study.**

| **GCN1L1 mRNA** | **F** | **TATGCCATGGACATCGTGAAAG** |
| --- | --- | --- |
|  | **R** | **TCTTCTGGGCTACAACAGTTAG** |
| **Circ****GCN1L1** | **F** | **GGAGGTTATGGGCAGGCT** |
|  | **R** | **TTCCGGATCTCCTCCTCC** |
| **MiR-330-3p** | **F** | **TATAGCAAAGCACACGGCCTGC** |
|  | **R** | **Universal Reverse Primer (Sangon Biotech)** |
| **U6** | **F** | **CTTCGGCAGCACATATACTA** |
|  | **R** | **AACTGGTGTCGTGGAGTC** |
| **β-actin** | **F** | **AAGGTGACAGCAGTCGGTT** |
|  | **R** | **TGTGTGGACTTGGGAGAGG** |
| **GADPH** | **F** | **AGCCACATCGCTCAGACAC** |
|  | **R** | **GCCCAATACGACCAAATCC** |
| **Human MMP3** | **F** | **TGACACACACTTTGAAGAGTAAC** |
|  | **R** | **TCACAGAGACTTAGGTGAAGAAT** |
| **Human MMP13** | **F** | **AGTGGTGGTGATGAAGATGATTTG** |
|  | **R** | **CATTTCTCGGAGCCTCTCAGTC** |
| **Human COL2A1** | **F** | **TCCTGCCGTTTCGCTG** |
|  | **R** | **CATTATACCTCTGCCCATCCTG** |
| **Human ADAMTS4** | **F** | **CCCGCTTCATCACTGACTT** |
|  | **R** | **ATGGAGCCTCTGGTTTGTCTA** |
| **Human TNF-α** | **F** | **AGAGGGAGAGAAGCAACTACAG** |
|  | **R** | **CGTGGGTCAGTATGTGAGAG** |
| **Human TNFR1** | **F** | **TCACCGCTTCAGAAAACCACC** |
|  | **R** | **GGTCCACTGTGCAAGAAGAGA** |
| **Human TNFR2** | **F** | **CGGGCCAACATGCAAAAGTC** |
|  | **R** | **CAGATGCGGTTCTGTTCCC** |
| **Human p65** | **F** | **ATGTGGAGATCATTGAGCAGC** |
|  | **R** | **CCTGGTCCTGTGTAGCCATT** |
|  |  |  |
| **Hsa-mir-330-3p mimics** |  | **GCAAAGCACACGGCCUGCAGAGA** |
| **Hsa-mir-330-3p inhibitor** |  | **UCUCUGCAGGCCGUGUGCUUUGC** |
|  |  |  |
| **CircGCN1L1 shRNA** |  | **AGTGGGAAGCCAGGGGCTG** |
| **CircGCN1L1 shRNA control** |  | **TTCTCCGAACGTGTCACGT** |
|  |  |  |
| **Probes for FISH** |  | **RiboBio** |
|  |  |  |
| **Primers of Luciferase assay reporter gene vector** | | |
| **CircGCN1L1 WT** | **F** | **AGATCGCCGTGTGACTCGAGGGGCTGATGGAACTCCACATG** |
|  | **R** | **GCCCCGACTCTAGCACGCGTCTGGCTTCCCACTGATCTGGA** |
| **CircGCN1L1 MT** | **F** | **AGATCGCCGTGTGACTCGAGGGGCTGATGGAACTCCACATG** |
|  | **R** | **GCCCCGACTCTAGCACGCGTCTGGCTTCCCACTGATCTGGA** |
| **TNF UTR WT** | **F** | **GAAAGATCGCCGTGTGACTCGAGGGAGGACGAACATCCAACC** |
|  | **R** | **CCGCCCCGACTCTAGCACGCGTTTTCTTTTCTAAGCAAACTTTATTTCTCG** |
| **TNF UTR MT** | **F1** | **GAAAGATCGCCGTGTGACTCGAGGGAGGACGAACATCCAACCTTCC** |
|  | **R1** | **GACCCTAGTGCTTAAAACTGTCGTTCTGGAGGCCCCAGTTTGA** |
|  | **F2** | **GACAGTTTTAAGCACTAGGGTCTCCCTGACATCTGGAATCTGG** |
|  | **R2** | **CCGCCCCGACTCTAGCACGCGTTTTCTTTTCTAAGCAAACTTTATTTCTCG** |

**Overexpression plasmid vector for circGCN1L1 (GM-10609).**

AATTATATTTATAAAAATAAATTTACAACTGTTTTGCAGATGGAACCTGAATTTTGGGACAAAAATCCCTTCAAGGCGACGGCCAAGGCTTTCCCACCAGGATTTCACTACAAACCAACAACCATCCTGAAGACAAGAACTTTTTATGAATTGATTTTGGTAGATTCAAACTCAGTATCTATCAAACATTTCAAAGACCCAAAAGACCAAACATTAAACACCCACTCGACAATCCAAATACTAAAAGTTCTTCAACCAAGACACTTTGGTTCGGACTTGAACAAAGGAAAGAGATTTTCAGTTCCATTTGATCCGGTAGGTTATACCTATTGGGATTATGTTGATGCTTGGACTAAGGTGTTTTGGCACCAAAATACCCGCTTCAAGCATTCATGGCTGATATATTTCAAGACCAATACAATTTACAATTTTCCCAATTGGTTTTTGCAATGGTGGGACTTCTTTGGACCAATCCCAGAGATATTCCCAGAAACAGTCCAACAAGGATTTGCTCAATTTGAGAGACAATACAATTCGCAGGAATCACGAATTCCAGCAGATCTTAAATACTTTTCAAGCTTTGCACTGTCATGGATCTTCTCATGGCAATATCGTTACAGCAAGACTGAAAAGACAAACCAATATCCATCACTACAAAGACACGCGTTTGTAAAATGGTGGACACAATTTGACTCATCCAAAGCGGATCCAGAACAAGTAAAACTCTGGTTCCAATCCCATCCAGAATTCCTCAAAGCCGCTGATCCAGAAACTTCTGTGTTTTTGAACCAAAAGTCTCATATGGCAGCATTTTTAGCAGGATCGAAATCAAAGGAGGTCTTAGCTAAAAATCTAAAGGAAGTTCTACAAATGTTACAGCAGGAAGAAGAAAGTTTATCCTCAAAGAAGGAAGAAACAAGTTCTGCTGAAGAAGAAGAAGAAGACCCCTTTTACCAAAACGAAGATGATTGTTTTGGTATCTGTTTAGATTAAATTTAATTTCGGTCACAAAACCGTCTGTTTGTAATTAATGTGTAATTAATGCTGGACAATACTACCTGTCCTGTAGCCATTAAAGTC

**ShRNA vector sequence (hsa_circ_0000448 shRNA (PGMLV-SC5)):**

ACAATACCTCAAAACGATACAGGCTGTTAGAGAGATAATTCGAATTCATTTGACTGTAAACACAAAGATATTAGTACAAAATACGTGACGTAGAAAGTAATAATTTCTTGGGTAGTTTGCAGTTTTAAAATTATGTTTTAAAATGGACTATCATATGCTTACCGTAACTTGAAAGTATTTCGATTTCTTGGCTTTATATATCTTGTGGAAAGGACGAGGATCCAGTGGGAAGCCAGGGGCTGCTCGAGCAGCCCCTGGCTTCCCACTTTTTTTAATTCTAGTTATTAATAGTAATCAATTACGGGGTCATTAGTTCATAGCCCATATATGGAGTTCCGCGTTACATAACTTACGGTAAATGGCCCGCCTGGCTGACCGCCCAACGACCTTCGCTCGATCAC

**Luciferase assay vector sequence information:**

**Overexpression vector for circGCN1L1 wild-type (PGL3-CMV-LUC-hsa_circ_0000448 WT):**

GGGCTGATGGAACTCCACATGGTATTGCCAGCACCTGATACTGATGAGAAGAATGGCCTGAACCTTCTGCGGAGACTCTGGGTGGTCAAGTTTGACAAGGAGGAGGAGATCCGGAAGCTGGCTGAGAGGCTCTGGTCAATGATGGGCCTAGACCTGCAGCCAGACCTCTGCTCCTTGCTGATTGACGACGTGATCTATCATGAGGCGGCTGTAAGGCAGGCAGGGGCCGAAGCCCTCTCCCAAGCAGTGGCACGTTACCAGCGGCAGGCGGCGGAGGTTATGGGCAGGCTCATGGAGATTTACCAGGAAAAGCTCTACCGGCCGCCCCCAGTGCTGGATGCTTTGGGACGAGTTATTTCAGAATCTCCTCCAGATCAGTGGGAAGCCAG

**Overexpression vector for circGCN1L1 mutant type (PGL3-CMV-LUC-hsa_circ_0000448 (hsa-miR-330-3p) MT):**

GGGCTGATGGAACTCCACATGGTATTGCCAGCACCTGATACTGATGAGAAGAATGGCCTGAACCTTCTGCGGAGACTCTGGGTGGTCAAGTTTGACAAGGAGGAGGAGATCCGGAAGCTGGCTGAGAGGCTCTGGTCAATGATGGGCCTAGACCTGCAGCCAGACCTCTGCTCCTTGCTGATTGACGACGTGATCTATCATGAGGCGGCTGTAAGGCAGGCAGGGGCCGAAGCCCTCTCCCAAGCAGTGGCACGTTACCAGCGGCAGGCGGCGGAGGTTATGGGCAGGCTCATGGAGATTTACCAGGAAAAGCTCTACCGGCCTAAAAACTGTAGTTCGTAGGGTTGACGAGTTATTTCAGAATCTCCTCCAGATCAGTGGGAAGCCA

**Overexpression vector for human TNF UTR WT (PGL3-CMV-LUC-H_TNF UTR WT):**

GGAGGACGAACATCCAACCTTCCCAAACGCCTCCCCTGCCCCAATCCCTTTATTACCCCCTCCTTCAGACACCCTCAACCTCTTCTGGCTCAAAAAGAGAATTGGGGGCTTAGGGTCGGAACCCAAGCTTAGAACTTTAAGCAACAAGACCACCACTTCGAAACCTGGGATTCAGGAATGTGTGGCCTGCACAGTGAAGTGCTGGCAACCACTAAGAATTCAAACTGGGGCCTCCAGAACTCACTGGGGCCTACAGCTTTGATCCCTGACATCTGGAATCTGGAGACCAGGGAGCCTTTGGTTCTGGCCAGAATGCTGCAGGACTTGAGAAGACCTCACCTAGAAATTGACACAAGTGGACCTTAGGCCTTCCTCTCTCCAGATGTTTCCAGACTTCCTTGAGACACGGAGCCCAGCCCTCCCCATGGAGCCAGCTCCCTCTATTTATGTTTGCACTTGTGATTATTTATTATTTATTTATTATTTATTTATTTACAGATGAATGTATTTATTTGGGAGACCGGGGTATCCTGGGGGACCCAATGTAGGAGCTGCCTTGGCTCAGACATGTTTTCCGTGAAAACGGAGCTGAACAATAGGCTGTTCCCATGTAGCCCCCTGGCCTCTGTGCCTTCTTTTGATTATGTTTTTTAAAATATTTATCTGATTAAGTTGTCTAAACAATGCTGATTTGGTGACCAACTGTCACTCATTGCTGAGCCTCTGCTCCCCAGGGGAGTTGTGTCTGTAATCGCCCTACTATTCAGTGGCGAGAAATAAAGTTTGCTTAGAAAAGAAA

**Overexpression vector for human TNF UTR MT (PGL3-CMV-LUC-H_TNF UTR MT):**

GGAGGACGAACATCCAACCTTCCCAAACGCCTCCCCTGCCCCAATCCCTTTATTACCCCCTCCTTCAGACACCCTCAACCTCTTCTGGCTCAAAAAGAGAATTGGGGGCTTAGGGTCGGAACCCAAGCTTAGAACTTTAAGCAACAAGACCACCACTTCGAAACCTGGGATTCAGGAATGTGTGGCCTGCACAGTGAAGTGCTGGCAACCACTAAGAATTCAAACTGGGGCCTCCAGAACGACAGTTTTAAGCACTAGGGTCTCCCTGACATCTGGAATCTGGAGACCAGGGAGCCTTTGGTTCTGGCCAGAATGCTGCAGGACTTGAGAAGACCTCACCTAGAAATTGACACAAGTGGACCTTAGGCCTTCCTCTCTCCAGATGTTTCCAGACTTCCTTGAGACACGGAGCCCAGCCCTCCCCATGGAGCCAGCTCCCTCTATTTATGTTTGCACTTGTGATTATTTATTATTTATTTATTATTTATTTATTTACAGATGAATGTATTTATTTGGGAGACCGGGGTATCCTGGGGGACCCAATGTAGGAGCTGCCTTGGCTCAGACATGTTTTCCGTGAAAACGGAGCTGAACAATAGGCTGTTCCCATGTAGCCCCCTGGCCTCTGTGCCTTCTTTTGATTATGTTTTTTAAAATATTTATCTGATTAAGTTGTCTAAACAATGCTGATTTGGTGACCAACTGTCACTCATTGCTGAGCCTCTGCTCCCCAGGGGAGTTGTGTCTGTAATCGCCCTACTATTCAGTGGCGAGAAATAAAGTTTGCTTAGAAAAGAAA
