# Supplementary material for: CircGCN1L1 promotes synoviocyte proliferation and chondrocyte apoptosis by targeting miR-330-3p and TNF-α in TMJ osteoarthritis
Source: Cell Death Dis. 2020 Apr 24;11(4):284. doi: 10.1038/s41419-020-2447-7 (PMC7181816; doi:10.1038/s41419-020-2447-7)
Supplement: Supplementary file 4 — Supplementary Table 3 [file 41419_2020_2447_MOESM4_ESM.docx]

| **Supplementary Table 3: Differentially expressed circRNAs** | | | | |
| --- | --- | --- | --- | --- |
| **CircRNAID** | **P-value** | **Fold Change** | **Regulation** | **Significance** |
| chr14:106090742-106109468- | 1.82424E-06 | 212.2674336 | up | Yes |
| chr6:29856288-29911088+ | 0.004039208 | 208.407598 | up | Yes |
| chr15:59204762-59209198- | 0.003997096 | 162.1749268 | up | Yes |
| chr12:120592774-120593523- | 0.004918223 | 153.3027674 | up | Yes |
| chr6:29856311-29911111+ | 0.003995295 | 124.5388113 | up | Yes |
| chr21:37711073-37717005+ | 0.005202956 | 104.7109689 | up | Yes |
| chr6:31236717-31321837- | 0.044190268 | 88.7187144 | up | Yes |
| chr9:96233423-96261168+ | 0.005748669 | 86.4664645 | up | Yes |
| chr6:32487269-32549455- | 0.005186404 | 70.6154375 | up | Yes |
| chr6:31238163-31323269- | 0.005563674 | 57.385023 | up | Yes |
| chr10:126370176-126370948- | 0.012512356 | 54.8238631 | up | Yes |
| chr8:131164982-131181313- | 0.047065301 | 47.7155196 | up | Yes |
| chr6:32522722-32549588- | 0.011014803 | 46.3768205 | up | Yes |
| chr2:198281465-198285266- | 0.038558029 | 37.794055 | up | Yes |
| chr2:61749746-61753656- | 0.041568209 | 37.3868022 | up | Yes |
| chr11:59426339-59426942- | 0.041641302 | 37.0421965 | up | Yes |
| chr6:33053556-33095875+ | 0.011654974 | 32.143515 | up | Yes |
| chr6:31239010-31324103- | 0.00246232 | 22.1762941 | up | Yes |
| chr12:70193989-70195501+ | 0.042616594 | 21.5977584 | up | Yes |
| chr6:31238984-31324077- | 0.016663632 | 21.2251597 | up | Yes |
| chr19:8520289-8528570+ | 0.040658798 | 20.6118584 | up | Yes |
| chr5:65349234-65350779+ | 0.040135172 | 19.1181169 | up | Yes |
| chr14:106090767-106134535- | 0.009790243 | 12.7771167 | up | Yes |
| chr14:106090742-106134510- | 0.010275534 | 12.5106929 | up | Yes |
| chr14:50320572-50329506- | 0.009210378 | 10.8911907 | up | Yes |
| chr11:62295775-62298156- | 0.006935239 | 7.6110594 | up | Yes |
| chr14:106110165-106208434- | 0.006313014 | 7.6110594 | up | Yes |
| chr1:52954581-52975384- | 0.005253019 | 7.0182181 | up | Yes |
| chr6:32522516-32523308- | 0.00462757 | 7.0182181 | up | Yes |
| chr14:106092177-106110900- | 0.043602127 | 4.4281963 | up | Yes |
| chr22:23243305-23264914+ | 0.04044292 | 4.4281963 | up | Yes |
| chr22:23243419-23261973+ | 0.04044292 | 4.4281963 | up | Yes |
| chr14:106207844-106235607- | 0.041972718 | 3.8549688 | up | Yes |
| chr2:72945232-72960247- | 0.001048419 | 425.3063321 | down | Yes |
| chr7:99090663-99092254- | 0.007037226 | 124.7929026 | down | Yes |
| chr5:134076753-134079742+ | 0.042656403 | 71.014036 | down | Yes |
| chr12:12397196-12397589- | 0.034954927 | 62.6837845 | down | Yes |
| chr1:58971732-59004982- | 0.033929272 | 56.5531574 | down | Yes |
| chr5:95091100-95099324+ | 0.013734778 | 55.0877881 | down | Yes |
| chr15:77471132-77474382- | 0.040988717 | 49.9975742 | down | Yes |
| chr3:196118684-196120490- | 0.042163744 | 49.6359038 | down | Yes |
| chr17:33495080-33495704+ | 0.03869255 | 47.1817659 | down | Yes |
| chr6:163876311-163899928+ | 0.040913862 | 46.2185396 | down | Yes |
| chr8:52773405-52773806- | 0.029720986 | 46.2026071 | down | Yes |
| chr1:172109620-172113577- | 0.033102505 | 45.0116447 | down | Yes |
| chr3:138289160-138291826- | 0.042768668 | 42.6529615 | down | Yes |
| chr1:104108057-104118162+ | 0.04383856 | 42.1416547 | down | Yes |
| chr18:8718422-8720494+ | 0.035893876 | 41.4628656 | down | Yes |
| chr2:152363423-152370942- | 0.038892225 | 39.8839342 | down | Yes |
| chr14:97026986-97029230+ | 0.033729349 | 38.5485807 | down | Yes |
| chr3:98568305-98600611- | 0.039076182 | 33.2732391 | down | Yes |
| chr5:64863340-64868113+ | 0.037052871 | 29.8949663 | down | Yes |
| chr6:34574332-34574681- | 0.044760317 | 21.2856896 | down | Yes |
| chr14:23863277-23893358- | 0.005788309 | 7.4848851 | down | Yes |
| chr4:100203547-100235021- | 0.046829312 | 6.2315651 | down | Yes |
| chr14:23863043-23893283- | 0.041520169 | 4.7226432 | down | Yes |
| chr1:196659238-196748971+ | 0.02385937 | 4.1112997 | down | Yes |
| chrX:139865340-139866824+ | 0.008757631 | 2.7296195 | down | Yes |
